# The landscape of tooth shape: Over 20 years of dental topography in primates

**DOI:** 10.1002/evan.21856

**Published:** 2020-07-20

**Authors:** Michael A. Berthaume, Vincent Lazzari, Franck Guy

**Affiliations:** ^1^ Division of Mechanical Engineering and Design London South Bank University London UK; ^2^ Department of Bioengineering Imperial College London London UK; ^3^ PALEVOPRIM—UMR 7262 CNRS INEE Laboratoire Paléontologie Evolution Paléoécosystèmes Paléoprimatologie Université de Poitiers Poitiers France

**Keywords:** dental topography, dental form, dental function, functional morphology, ecomorphology

## Abstract

Diet plays an incontrovertible role in primate evolution, affecting anatomy, growth and development, behavior, and social structure. It should come as no surprise that a myriad of methods for reconstructing diet have developed, mostly utilizing the element that is not only most common in the fossil record but also most pertinent to diet: teeth. Twenty years ago, the union of traditional, anatomical analyses with emerging scanning and imaging technologies led to the development of a new method for quantifying tooth shape and reconstructing the diets of extinct primates. This method became known as dental topography.

## 1 MOLAR SHAPE, SIZE, AND DIET

Anyone who studies dental evolution is undoubtedly familiar with George Cuvier's famous quote, “Show me your teeth and I will tell you who you are,” (translated from French).^1^, ^36^ Dental form (shape + size) is highly genetically controlled and well reflects phylogenetic ancestry. This makes teeth useful for systematics. Teeth are also adapted to diet in animals—particularly ones that chew their foods—and can be used to reconstruct aspects of dietary ecology.^2^, ^3^ In this respect, Cuvier's quote could be adapted to say, “Show me your teeth, and I will tell you what you are adapted to eat.”

**FIGURE 1:**
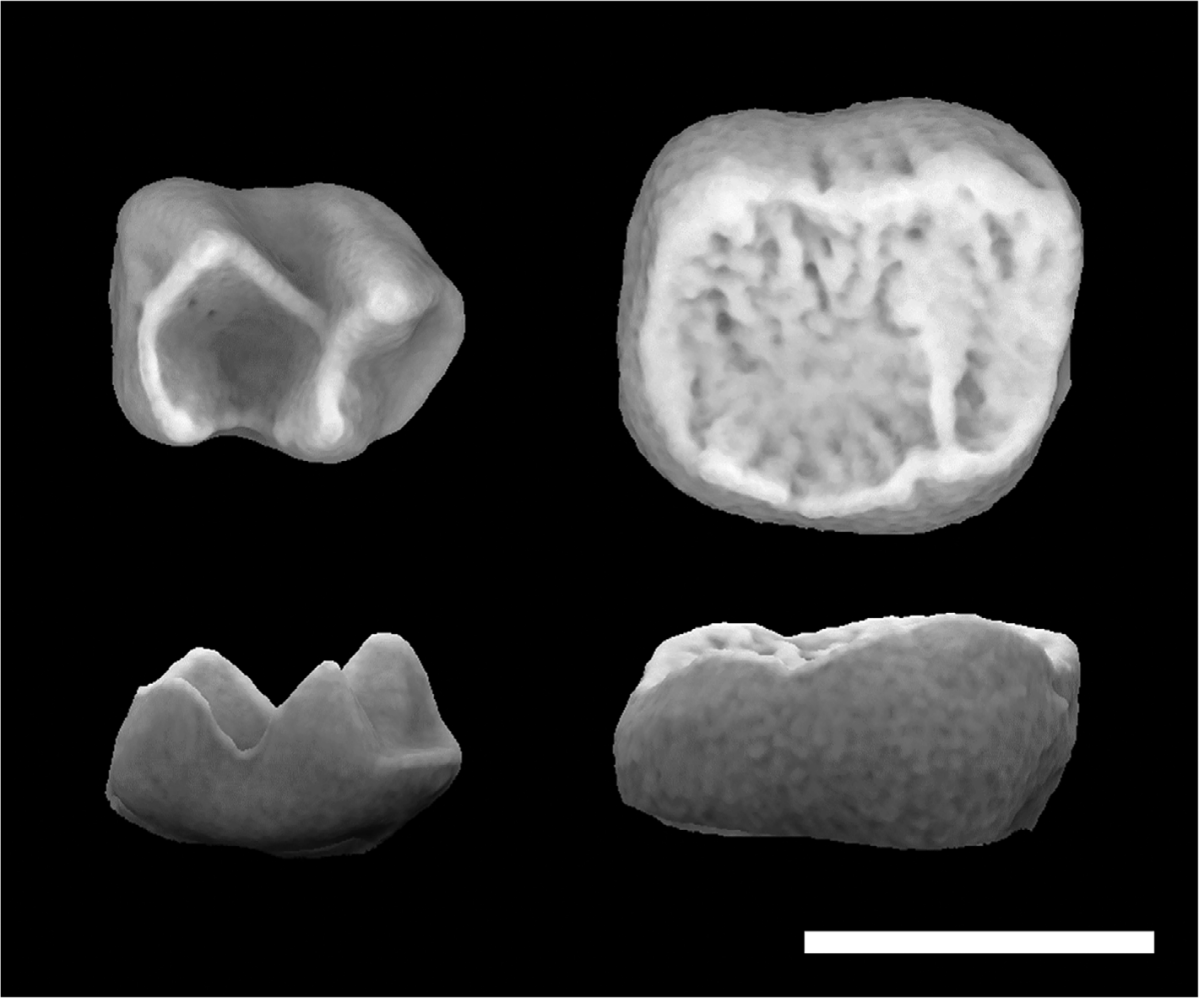
Occlusal and lateral views of *Galago alleni* (left, AMNH‐236348) and *Pithecia pithecia* (right, USNM‐374746, morphosource.org, reflected) M2s. Note the taller, sharper cusps on the *Galago* molar and crenulated surface of the pithecine molar. Scale = 3 mm

In primates, like other mammals, there is a strong relationship between tooth shape, size, and diet. For example, galagos have sharp, pointy molar cusps, which are efficient at piercing/crushing insects and cutting chitin into smaller pieces. This increases the food's digestibility and calories that can be obtained from the chitin.^4^ Conversely, pithecines have crenulated, bunodont molars with short, dull cusps, which are efficient at gripping nuts and maintaining structural integrity in the presence of high bite forces (Figure 1).^4^, ^5^, ^6^, ^7^ Applying this knowledge to the fossil record, it is easy to use gross dental morphology to make broad conclusions about the diets of extinct primates, such as whether a primate was primarily frugivorous or folivorous. However, it is difficult to use dental form to ask more refined dietary questions—such as whether primary or fallback foods played a larger role in dental evolution—that reveal more about primate ecology and evolution—such as interspecies competition—without quantifying form first.

The definition of diet changes depending on the question being asked. When discussing tooth shape and diet, it is often defined in two ways: First, using mechanical aspects of the foods consumed (e.g., how hard, soft, or tough the foods being consumed are), as the mechanical interactions between the foods and teeth are hypothesized to exert a large selective pressure on dental form,^8^, ^9^ or second, in terms of broad, ecologically defined dietary categories (e.g., folivory, frugivory, omnivory).^6^, ^10^ In these cases, it is often assumed that there is a relationship between the mechanical and ecological aspects of diet (e.g., leaves need to be sheared, and fruits need to be crushed),^11^ which is why there is a relationship between ecological diet and dental form. Occasionally, the two categories are combined, often to investigate the adaptations of hard object feeding (i.e., durophagy).^6^, ^12^

### 1.1 Dental form and function

**FIGURE 2:**
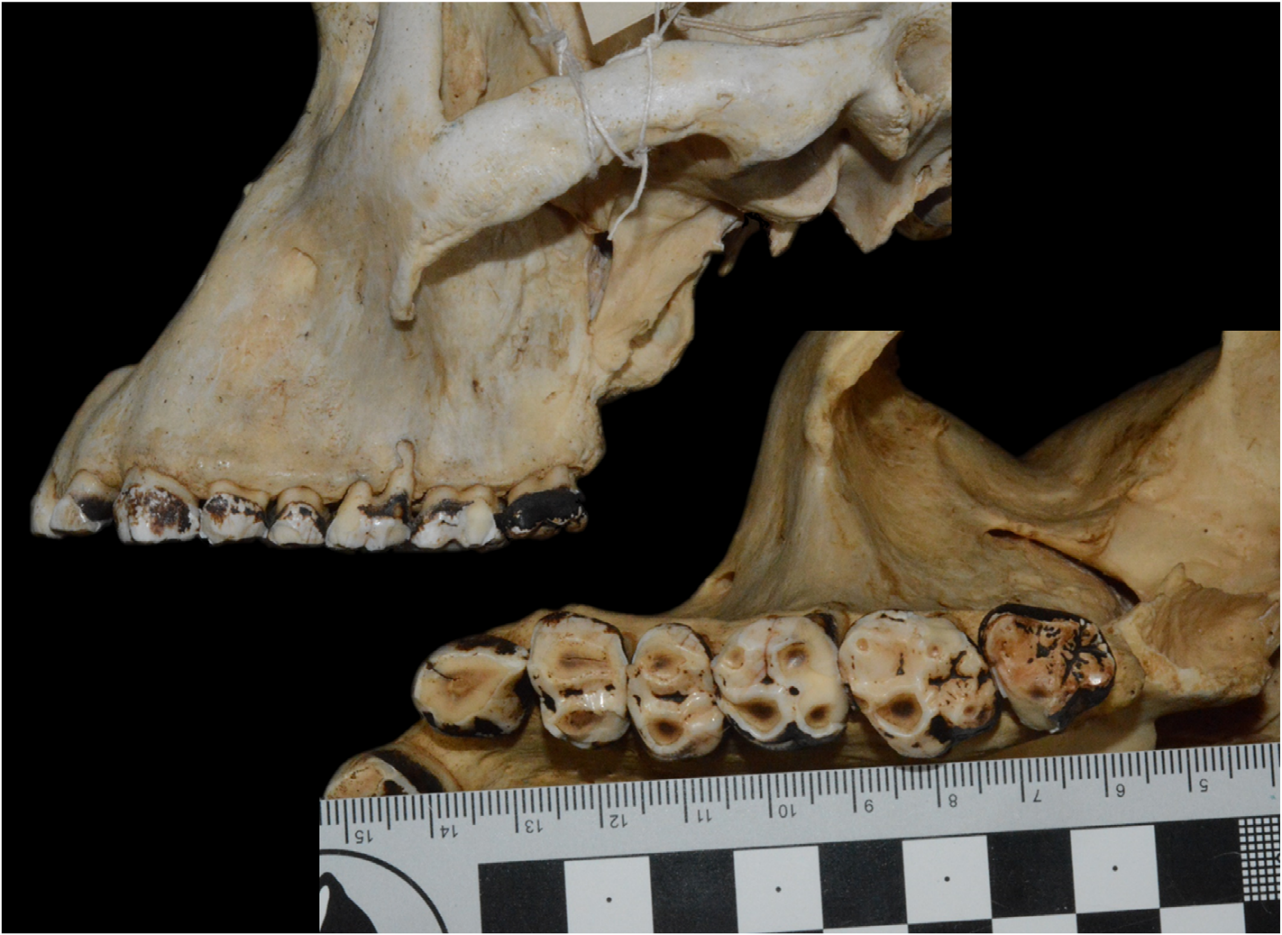
Female *Gorilla beringei beringei* specimen (accession ID 630739, Natural History Museum, Stockholm) with an upper canine that has functionally become part of the chewing row

Primate teeth are multifunctional tools and play an important role in food item breakdown. During feeding, incisors and (sometimes) canines are used to ingest foods, dividing foods into pieces small enough to fit in the oral cavity.^13^, ^14^ Premolars and molars are used to masticate foods by shearing, crushing, and grinding them in the oral cavity.^9^, ^15^ Exceptions include strepsirrhines with toothcombs, which do not use their lower incisors/canines to parse foods or their caniniform premolars (P_3_) to chew foods, and some hominoids, which can wear their canines to the level of the postcanine tooth row, making them “masticatory teeth” (Figure 2: Box 1). Because incisors and canines serve several nondietary functions, such as communication, their form is a result of dietary and nondietary selective pressures. This weakens the correlation between incisor/canine form and diet. However, the monofunctional role of postcanine teeth (food breakdown) has created a strong relationship between molar form and diet.

BOX 1 **Choice of tooth**

The first topographic studies used M_2_s, and many subsequent analyses followed suit. But, why M_2_s and not the entire postcanine tooth row, as in Evans and colleagues?^50^

The use of M_2_s can be traced to two studies, which use the second to last tooth in the dental row, as it was the most “average”‐shaped molar.^19^, ^20^ Some studies maintain this protocol, using M_1_s when M_3_ is absent, while others use M_2_ for homology. Lower molars are used because, under the mortar and pestle hypothesis, lower molars act as a pestle, breaking foods, while upper molars act as a mortar, stabilizing them.^11^, ^15^, ^83^ Therefore, lower molar shape should reflect food item breakdown, while upper molar shape should reflect food item stabilization. A study comparing RFI, OR, and SQ in platyrrhine upper and lower M^1^s supports the preferential use of lower molars for dietary reconstruction, while pointing toward the usefulness of upper molars.^62^ Third molars are more variable in shape, but Glowacka and colleagues found M^3^s gave similar results as M^1^s and M^2^s in known age mountain gorillas.

Using the entire tooth row can be problematic. First, not all specimens have the entire tooth row preserved. Second, dental topography is sensitive to tooth wear,^40^, ^42^, ^47^, ^63^, ^65^, ^66^, ^67^ and differences in timing of dental eruption cause variable levels of wear between teeth within a chewing row (e.g., M^1^ vs. M^3^, Figure 2). This can be exacerbated by differences in dental wear rates due to diet. In these cases, it is not possible to hold wear stage. Finally, there is sometimes a problem in deciding which teeth should be considered part of the chewing tooth row, and how to hold that constant between species. In some strepsirrhines, the caniform LP_3_ is not part of the chewing row, and some primates incorporate their canines into their chewing row (Figure 2). Further, what if third molars are not present in only some of the sample (e.g., callitrichids—marmosets, tamarins), or when supernumerary teeth are present, like fourth molars?^109^ While tooth rows present a more comprehensive picture, they can be much more problematic. That being said, more information is needed to investigate variation in dental topography along the tooth row. In particular, information on premolar tooth shape is needed, as this could reveal novel aspects of primate dental adaptations.^54^, ^110^

**FIGURE 3:**
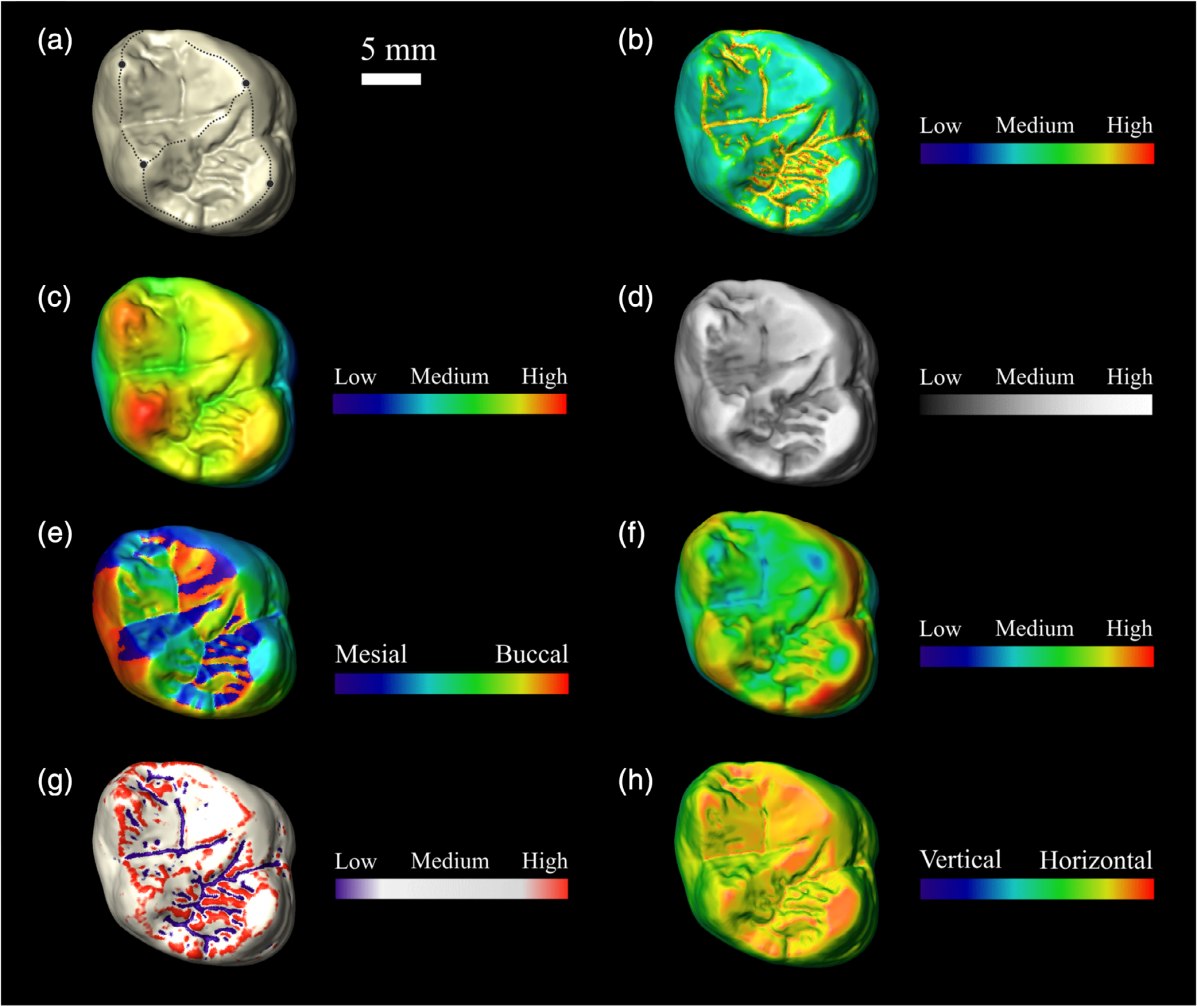
Occlusal views of a *Gorilla gorilla* second upper molar (MRAC‐27755) displaying (a) shearing crests for SQ calculation, and morphometric maps for (b) DNE, (c) elevation, (d) PCV, (e) OPC, (f) enamel thickness, (g) mean curvature, and (h) inclination. Scale bar is 5 mm. DNE, Dirichlet normal energy; OPC, orientation patch count; PCV, portion of visible sky; SQ, shearing quotient

Kay and colleagues developed one of the first metrics for quantifying primate occlusal molar shape (herein, tooth shape) in a dietary context, correlating M_2_ shearing capability to chewing efficiency (the ability to break down foods^16^, ^17^, ^18^, ^19^).^4^, ^17^, ^19^ In their experiments, insectivores, with relatively longer shearing crests, had higher chewing efficiencies than frugivores, with relatively shorter shearing crests (Figure 3).^4^, ^17^, ^19^ They hypothesized that primates with diets difficult to digest (e.g., chitin in insects, fiber in leaves) evolved relatively longer shearing crests, allowing them to digest food more efficiently. Their measure for shearing capability evolved into the shearing quotient (SQ: Box 2).^20^, ^21^, ^22^ The SQ is determined by regressing shearing crest length, the sum of a set of linear distances between discrete, homologous, and anatomical landmarks on the occlusal surface, against tooth length. Primates with positive residuals have relatively longer shearing crests and negative residuals have relatively shorter crests. In this respect, SQ analyzed tooth shape while accounting for allometric differences in tooth size.

BOX 2 **Glossary of abbreviations**

**Ambient occlusion** (portion de ciel visible, PCV: translated to “portion of visible sky”): A dental topographic metric that utilizes a computer graphics technique to make surfaces appear 3D by approximating the proportion of ambient light shining on a surface to quantify a tooth's morphological wear resistance (i.e., how effective the shape of the tooth is at resisting wear).

**Basin cutoff** (BCO): Method for cropping digital representations of a tooth, where only the portion of the tooth superior to the inferiormost point in the occlusal basin is considered.

**Dental topography** (DT) or dental topographic analysis (DTA): A landmark free method of quantifying and representing 2.5 or 3D whole tooth shape with a single metric.

**Dirichlet normal energy** (DNE): A dental topographic metric that quantifies the curvature of a surface using Dirichlet energy. Within primates, teeth with curvy surfaces are generally sharper: as such, DNE is often used to quantify tooth sharpness.

**Entire enamel cap** (EEC): Method for cropping digital representations of a tooth, where the entire outer surface of the enamel cap is considered.

**Enamel‐dentin junction** (EDJ): The boundary between the enamel and the underlying dentin in a tooth.

**Finite element analysis** (FEA): Method for solving engineering and mathematical models using a meshed area of interest, constitutive equations, boundary conditions, and material properties.

**Geographic information systems** (GIS): Conceptual framework that provides the user with the ability to capture and analyze spatial and geographic data.

**Micro‐computed tomography** (microCT): An imaging technique where X‐rays are used to take slice‐by‐slice images of an object, and computer algorithms are used to reconstruct the 3D object.

**Outer enamel surface** (OES): The portion of the enamel cap that is exposed to the external environment.

**Orientation patch count** (OPC): A dental topographic metric that quantifies the orientation of each polygon on a digitized tooth's surface and counts the number of “patches” that form on the tooth, where a patch is defined as a predetermined number (often 3 or 5) of adjacent polygons with the same orientation. It is used to estimate dental complexity.

**Orientation patch count rotated** (OPCR): A derivative of OPC that normalizes for initial error in tooth orientation by rotating an occlusally aligned tooth clockwise or counter‐clockwise (usually 8 times), calculating OPC at each new orientation, and averaging all the OPC values together.

**Occlusal relief** (OR): A dental topographic metric that quantifies the relative height of the occlusal portion by first cropping a tooth using the basin cutoff (BCO) method, and then taking the ratio of the tooth's outer enamel surface (OES) area to its cross‐sectional area.

**Relief index** (RFI): A dental topographic metric that quantifies the relative height of a tooth by taking the ratio of a tooth's outer enamel surface (OES) area to its cross‐sectional area. It differs from occlusal relief (OR) in that RFI utilizes the entire enamel cap (EEC).

**Shearing ratio** (SR): A derivative of the shearing quotient, which calculates the relative length of a shearing crest in a manner independent of the sample being analyzed.

**Shearing quotient** (SQ): A dental topographic metric that quantifies the relative length of a shearing crest on a tooth's surface. As it utilizes residuals, SQ metrics are dependent on the sample being analyzed.

Later, researchers used the SQ, and derivatives thereof, such as the shearing ratio (SR) and shearing ratio based on body mass (SRM),^6^, ^23^, ^24^ to show that folivores also have relatively long shearing crests, presumably because of their high‐fiber diets.^22^, ^25^, ^26^, ^27^ Although primates that are primarily insectivorous and folivorous have similar relative shearing crest lengths, it is possible to differentiate between them using body size: insectivorous primates are ≤250 g and folivorous primates are ≥700 g.^28^ Together, this research showed that insectivores and folivores have relatively longer shearing crests than frugivores and hard‐object feeders (i.e., durophages). This may be because the selective pressure acting on chewing efficiency is stronger in insectivores and folivores than the selective pressure acting on fruit smashing/juicing^11^, ^15^ and dissipating high bite forces,^29^, ^30^, ^31^, ^32^ and the opposite is true for frugivores and hard‐object feeders.

**FIGURE 4:**
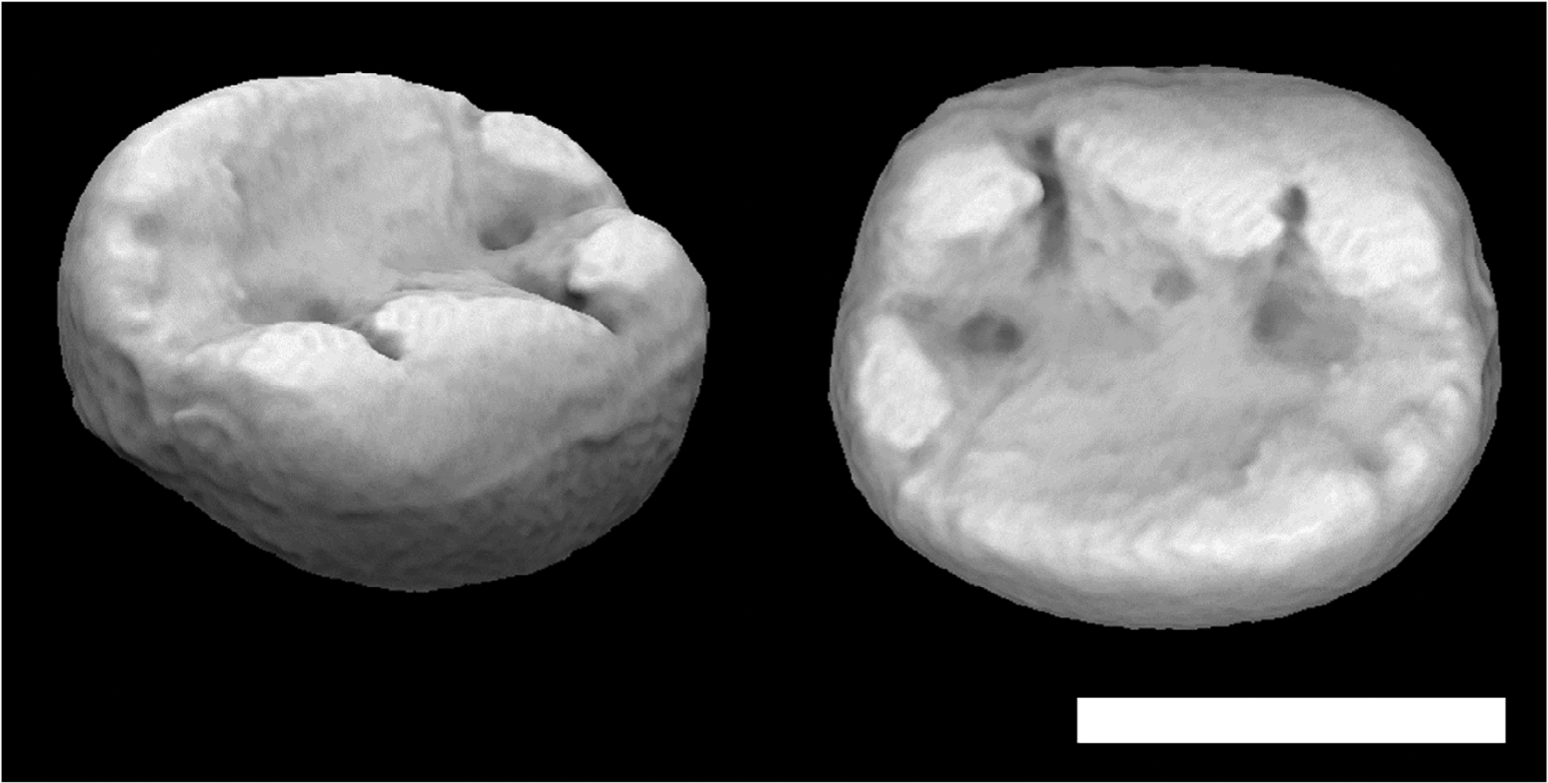
*Daubentonia madagascariensis* M2 (AMNH‐41334, morphosource.org). Scale = 3 mm

Despite successes, these metrics were limited by their reliance on occlusal landmarks that could only be measured on relatively unworn teeth with prominent shearing crests. This prevented the inclusion of molars that were worn and taxon with poorly developed molar shearing crests (e.g., *Daubentonia*, Figure 4) from topographic analyses.^6^ Importantly, complex ecological questions related to dental wear could not be addressed. For example, what are the effects of climate change on primate dietary ecology?^33^ As global warming changes the environment and thereby food availability, what is the likelihood different species will survive, or go extinct?^33^ How does climate/climate change and consumption of invasive species affect dental wear, evolutionary fitness, and primate evolution?^27^, ^34^, ^35^ How does tooth shape change throughout an animal's life, and how does this affect its ability to survive? And finally, how is tooth shape affected by factors such as primary/fallback foods and foods with different physical properties, and how does that correlate with an animal's ability to survive?^8^, ^9^

To address more complicated questions about dental ecology,^36^ a new method needed to be developed. But first, barriers related to data acquisition and quantification had to be overcome.

### 1.2 The development of dental topography

The first barrier was how to digitally capture whole tooth shape. Previously, whole tooth shape did not need to be captured, as shearing crest length was measured using linear distances and a microscope reticle.^22^ But for whole tooth shape to be quantified, it needed to be captured.

The first attempt used a low‐resolution electromagnetic 3D scanner to produce a rough digital approximation of the occlusal surface.^37^ A later attempt used laser confocal microscopy:^38^ this produced more accurate scans, but did not gain traction in primate dental studies. Eventually, laser and micro‐computed tomography (microCT) scanners were chosen as effective ways of creating digitized representations of teeth.^10^, ^39^

The second barrier was how to quantify tooth shape without landmarks.^40^ Most studies came to the same conclusion: if cusps were treated as mountains and basins as valleys, geographic information systems (GIS) software, developed to quantify landscape topography, could be used to quantify tooth shape.^37^, ^38^, ^39^ The idea of using GIS software to quantify tooth shape was a novel,^1^ clever way of excluding landmarks, allowing for the quantification of worn tooth shape.^40^ This new method for quantifying tooth shape was dubbed dental topography.

## 2 DENTAL TOPOGRAPHY DEFINITIONS

**FIGURE 5:**
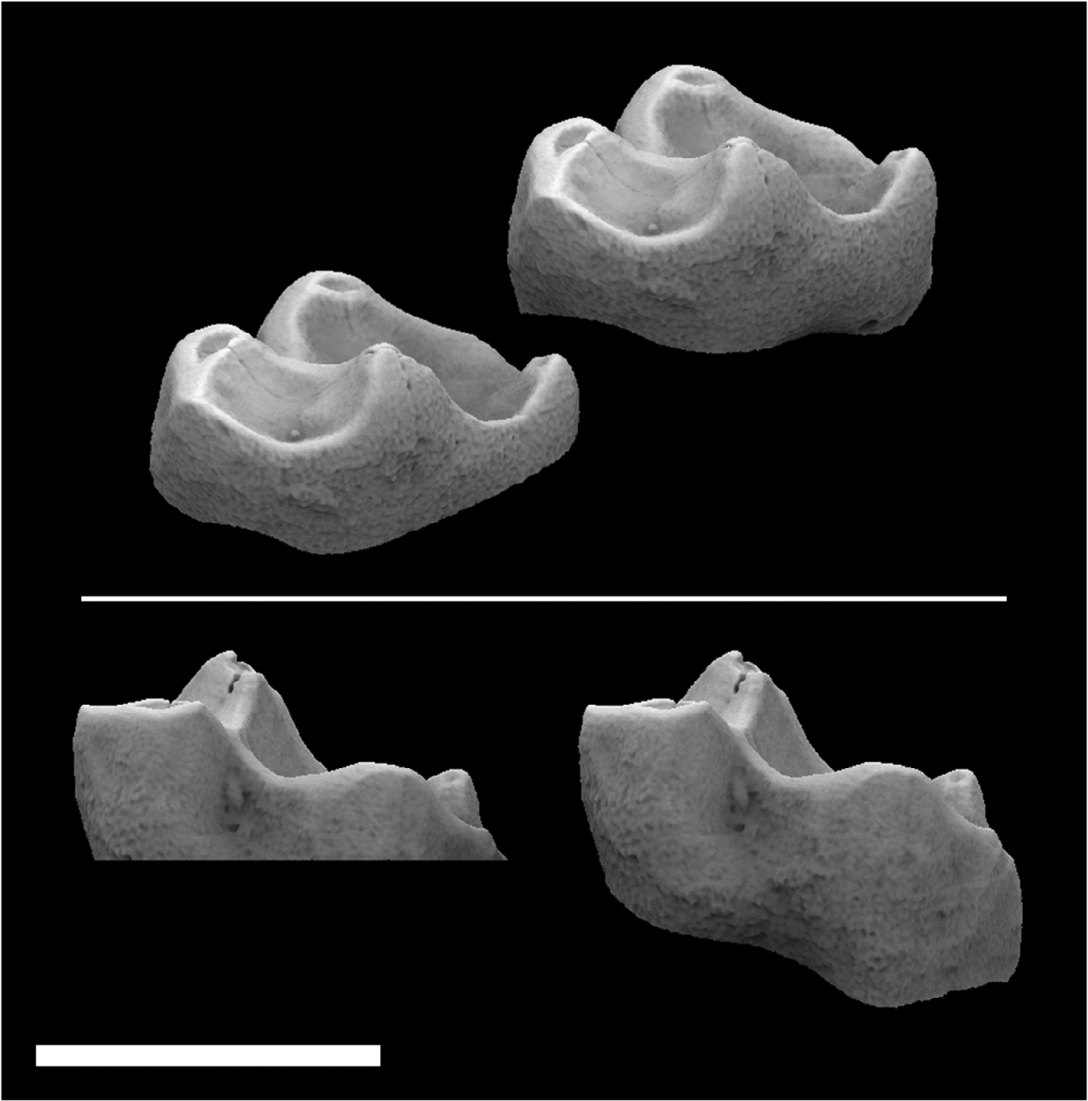
*Alouatta palliata* tooth (USNM 171063, morphosource.org) cropped using the BCO (left) and EEC (right). Scale = 6 mm. BCO, basin cutoff; EEC, entire enamel cap

The term “dental topography” gained its present meaning in 2000, where it was defined as “a method for modeling the shapes of the biting surfaces of teeth as topographic surfaces for analysis using geographic information systems technology.”^39^ Since 2000, studies have incorporated more aspects of the tooth than just the biting surface (e.g., enamel walls) and used non‐GIS software and techniques.^10^, ^41^, ^42^, ^43^ As such, Berthaume^44^ suggested defining dental topography as, “a [landmark free] method of quantifying and representing 2.5 or 3D whole tooth shape with a single metric.”^44^ Importantly, both definitions exclude landmark‐based metrics like SQ. Although exact procedures vary, all topographic studies have the same underlying protocol, involving tooth digitization, digital preprocessing/editing, and shape quantification (Figure 5; Box 3).

BOX 3 **Performing dental topographic analyses**

The following steps are consistent across all topographic studies:

1. Obtain specimens or molds of teeth from collections.
2. Take 2.5D or 3D scans of the teeth.
3. Edit scans to isolate portions of the tooth for quantification.
4. Quantify tooth shape using one or more parameters.

Scanning original material is preferential, but not always possible. If scanning original material with laser or light scanners, enamel may need to be coated with a mat substance (e.g., Magnaflux Spotcheck SKD‐S2 Developer) to reduce the reflectivity of the enamel.^54^, ^67^

Topographic analyses use 2.5D and 3D scans. 2.5D scans are projections of a 2D plane into the third dimension, meaning one height coordinate exists for each pair of length and width coordinates. This generally represents the occlusal surface well, but portions of the tooth remain hidden,^39^, ^40^, ^63^ preventing the calculation of some topographic metrics (e.g., RFI). Tactile, laser, and light scanners typically generate 2.5D scans. 3D scanners (e.g., microCT,^10^ X‐ray synchrotron microtomography)^111^ are generally more expensive, but capture all aspects of tooth shape.^6^, ^10^ Scans are either output as point clouds or surface (polygon) files.

Tooth orientation is important, particularly when taking 2.5D scans or when using orientation‐sensitive metrics (e.g., OPC, RFI).^74^ Teeth are generally oriented in anatomical position (i.e., how it would be in the mouth),^10^, ^40^, ^42^, ^54^, ^63^ maximal occlusal view,^27^, ^50^ or using the tips of dentin horns.^43^ The first two methods suffer from human error, and the last suffers the use of landmarks and internal geometry. The last method also risks orienting the tooth in a physiologically unrealistic manner, particularly if there is high variation in cusp height, as such, the authors recommend not using this method.

After scanning, surfaces are edited, cropped, and smoothed using a variety of programs (e.g., ArcMap,^112^ Avizo,^10^, ^43^, ^55^ Geomagic,^42^, ^43^, ^55^ Meshlab,^41^ and CloudCompare^54^). The two most popular cropping methods are the basin cutoff (BCO) and the EEC.^42^ BCO isolates the portion of the tooth superior to the inferiormost point in the occlusal basin (Figure 5). A drawback to this method is some molars have deep basins and mesially‐inclined cervical margins, so the BCO results in the inclusion of portions of the tooth root.^10^ Further, variable percentages of the enamel cap are deleted, particularly when teeth are worn and have deep dentin pools.^27^ The EEC method analyzes portion of the entire tooth, and not just portions responsible for food item breakdown. Teeth cropped using these two methods cannot be directly compared.^42^

Studies have investigated the sensitivity of EEC to cropping around the cervical margin^10^, ^41^, ^74^ have revealed topographic parameters are insensitive to intra‐ and inter‐observer error. However, larger samples size need be considered.

During editing, scans are normalized by resolution or triangle count, as some topographic metrics are sensitive to triangle count (e.g., curvature, DNE, OPCR).^33^, ^49^, ^76^ There appears to be no ideal triangle count for dental topographic analyses,^33^, ^41^, ^54^, ^55^, ^73^, ^76^ but resolution/triangle count must be high enough to represent the surface.

As with editing and cropping, there is no ideal smoothing method. Some topographic metrics, such as RFI, are relatively insensitive to smoothing, while others, like DNE, are sensitive to smoothing and smoothing protocol.^42^, ^49^, ^54^ *There are many acceptable methodologies for performing dental topographic analyses, and none are perfect*; but if methodologies are consistent, measures are comparable.

The main topographic metrics used today and their mathematical and biological meanings are presented in Table 1 and briefly discussed in the following.

**TABLE 1:** Dental topographic metrics currently in use

Metric	Paper introduced	Computational meaning	Biological meaning	Computer programs	Notes
Relief index (RFI, OR)	^35^, ^37^	Ratio of 3D surface area to 2D projected area	Relative crown height	Morphotester,^45^ molaR,^46^ Avizo + ImageJ, ArcGIS	RFI^36^ when the EEC cropping method is used, OR^35^ when BCO^1^, ^47^ is used
Slope	^35^	The average change in elevation		ArcGIS	Similar to inclination
Angularity	^35^	The average change in slope	Tooth sharpness	ArcGIS	Similar to curvature
Shearing crest length (2D and 3D)	^28^, ^48^	Length of border between patches that faces primarily buccal to primarily lingual	Shearing crest	GRASS GIS	
Orientation patch count (OPC)	^49^	Sum of the changes in triangle patch direction	Complexity; number of “tools” on the occlusal surface	Surfer, Morphotester,^45^ molaR^46^	OPC/OPCR metrics calculated from 2.5 and 3D scans are not comparable
Dirichlet normal energy (DNE)	^39^	Variability in surface curvature	Tooth curviness or sharpness	Morphotester,^45^ molaR,^46^ Teether	
Orientation patch count rotated (OPCR)	^50^	Average OPC over eight orientations	Complexity; number of “tools” on the occlusal surface	Surfer, Morphotester,^45^ molaR^46^	A way of normalizing OPC for tooth orientation
Elevation	^41^	*z*‐coordinate corresponding to each polygon	Absolute tooth height	R	
Inclination	^41^	The angle between the vector normal to the polygon's surface in the *z*‐direction and the horizontal *xy* plane		R	Similar to slope
Curvature	^41^	Deviation of flatness of the tooth surface	Tooth sharpness	R	Similar to angularity
Orientation	^41^	Direction of the polygon normal vector	Complexity; number of “tools” on the occlusal surface	R	Similar to OPC/OPCR
Ambient occlusion (portion de ciel visible, PCV)	^40^, ^42^, ^51^	Estimation of how much light is shining on a point on the surface	Morphological wear resistance	CloudCompare	

*Note*: Others (e.g., cusp and basin volume) have been, but are no longer used. An additional program, Dental Toolkit, will soon be available for dental topographic analysis.

### 2.1 Ambient occlusion (portion de ciel visible: translated to “portion of visible sky”)

**FIGURE 6:**
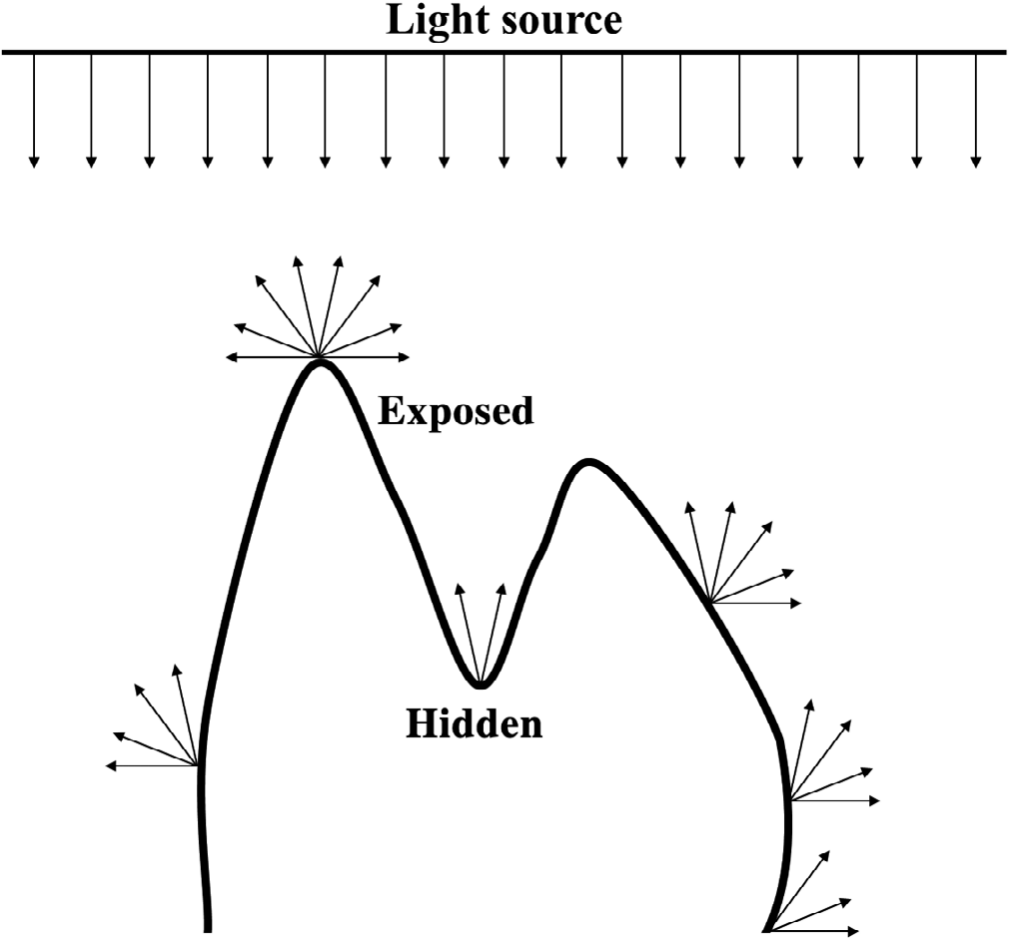
*Alouatta palliata* tooth (USNM 171063, morphosource.org) cropped using the BCO (left) and EEC (right). Scale = 6 mm. BCO, basin cutoff; EEC, entire enamel cap

Ambient occlusion is a computer graphics technique used to make surfaces appear 3D by approximating the proportion of ambient light shining on the surface. The specific method for ambient occlusion being discussed here is portion de ciel visible (“portion of visible sky,” PCV). If a tooth is oriented as if it were positioned in situ within a maxilla/mandible and light is shone from the occlusal direction, points on the tooth that interact more with the bolus/occluding tooth during a masticatory cycle (e.g., cusps, crests) tend to have higher ambient occlusion values, and points that interact less with the bolus/occluding tooth during a masticatory cycle (e.g., basins, enamel walls) tend to have lower ambient occlusion values (Figure 6). As PCV values are normalized between zero and one, they can be thought of as probabilities that portions of the tooth will interact with the bolus/occluding tooth during a given masticatory cycle. This provides location‐specific information about which parts of the tooth are more/less likely to contact the bolus/occluding tooth, and thereby experience wear. Average PCV has, therefore, been suggested a measure of morphological wear resistance (i.e., how effective the shape of the tooth is at resisting wear).

A study testing the relationship between PCV and diet in platyrrhines and prosimians has shown primates with lower crowned teeth and/or teeth with bulbous cusps, like those found in frugivores and hard‐object feeders, have higher average PCV, and primates with higher crowned teeth and/or teeth with taller cusps, like those found in folivores and insectivores, have lower average PCV.^52^ This was supported by another study on South African hominins, which showed a strong relationship between relative crown height and PCV in *Homo naledi*, *Paranthropus robustus*, and *Australopithecus africanus*.^42^ Interestingly, PCV appears efficient at predicting what spots of a tooth will experience wear once wear facets have formed.^52^ As dental wear occurs from dietary and environmental sources, it is possible PCV could be used to address questions concerning dietary and environmental shifts.

### 2.2 Angularity and curvature

These metrics quantify the sharpness of a tooth's surface. Mathematically, angularity is the second derivative of elevation (i.e., the change in slope across the surface), and the inverse of the second derivative of elevation is sharpness, so lower angularity values correspond to sharper teeth.^40^, ^53^ Curvature is similar, but calculated by taking the mean of the two principal curvatures for each polygon used to digitally represent the surface of the tooth.^43^ Essentially, it measures how much the tooth's surface bends at different points on the surface—areas that bend more are sharper.

Teeth with sharper occlusal surfaces, like those found in species with relatively long shearing crests, tend to have higher angularity and curvature than species with relatively shorter shearing crests.

### 2.3 Dirichlet normal energy

The variability in any mathematical function can be quantified using Dirichlet energy. Functions that are more curvilinear tend to be more variable and have higher energy. Dirichlet normal energy (DNE) measures surface variability, meaning teeth with higher DNE have curvier, or more variable, surfaces. Within primates, teeth with curvy surfaces (e.g., those with lots of cusps and crests or crenulations) are generally sharper.^41^ Primates with relatively taller cusps and crenulated surfaces have higher DNE than those with relatively shorter cusps.^6^, ^42^, ^54^, ^55^

**FIGURE 7:**
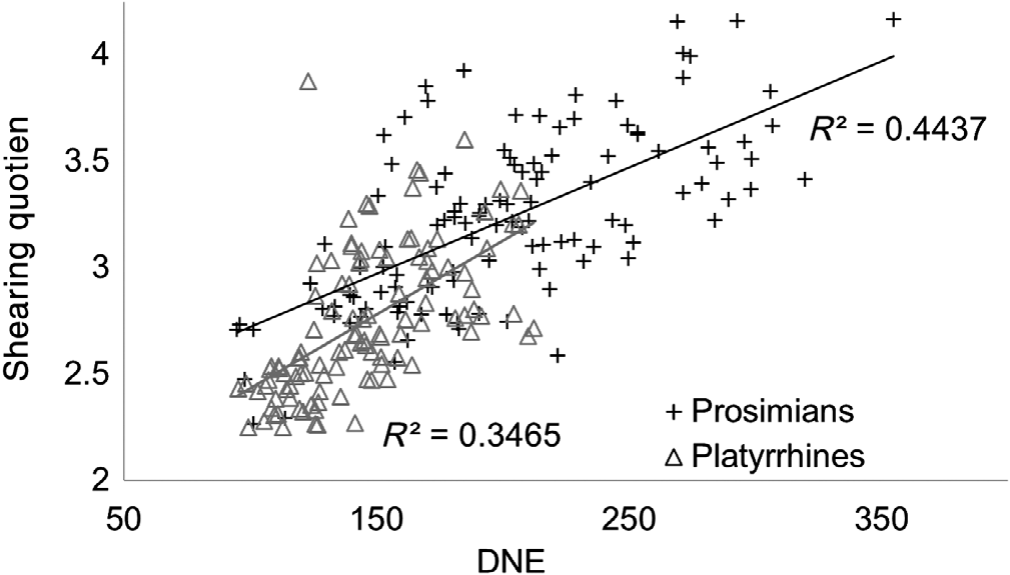
SQ versus DNE for prosimians (black crosses) and platyrrhines (gray triangles). Pearson's *R* ^2^ = 0.4437 for prosimians and 0.3465 for platyrrhines. Data from Reference 7. DNE, Dirichlet normal energy; SQ, shearing quotient

DNE is conceptually and geometrically similar to angularity,^45^ curvature, and SQ. However, a recent study showed DNE and angularity are poorly correlated^56^ and the correlation between SQ and DNE is weak (Figure 7), meaning that, although these metrics are similar, they are not interchangeable or directly comparable. It is therefore possible for studies that use DNE, angularity, and SQ to reach different conclusions, even though they quantify similar aspects of dental function.

When calculating DNE, a percent of the data can be discarded to account for geometrical singularities (e.g., sharp points/edges) that artificially inflate the score,^46^ usually 0.1% area × energy. A larger percentage (1–5%) may be discarded when many geometrical singularities are present (e.g., due to taphonomic erosion, scanning artifacts).^42^ Contour DNE plots on the tooth's surface can help determine if this is needed.^49^ Different DNE programs (e.g., the R package molaR^57^ and morphotester)^46^ have different protocols for excluding triangles at the edge of the surface. Excluding a variable number of triangles can be problematic, as DNE is sensitive to triangle count (see Box 3).^49^, ^52^ A newly introduced metric, ariaDNE, appears to be less sensitive to these factors compared to DNE.^58^

### 2.4 Elevation

Elevation is a height map of the tooth: it has yet to be correlated to diet.^43^, ^50^ It is useful in quantifying absolute tooth and/or cusp height.

### 2.5 Orientation, orientation patch count, and orientation patch count rotated

These metrics estimate dental complexity. Complexity can be thought of as the number of locations on the tooth's surface where foods are likely to fracture, and is presumably correlated to the number of occlusal features (i.e., cusps, crests, crenulations). Orientation patch count (OPC) quantifies complexity by calculating the normal vector of each triangle on the tooth's surface and binning triangles into eight categories depending on which (inter)cardinal direction the vector is pointing (up = N, right = E, down‐left = SW, etc.). If two or more triangles share an edge and are binned in the same category, they form a patch. OPC sums the number the patches with at least *X* triangles on the surface, where *X* is defined by the user. Often, *X* has values of 3 or 5.

In general, mammalian herbivores have postcanine tooth rows with higher complexity than carnivores.^50^ This pattern can be elucidated from a single molar, with mandibular teeth predicting diet better than maxillary ones.^48^, ^59^ Orientation patch count rotated (OPCR) is a way of normalizing for initial error in tooth orientation by rotating an occlusally‐aligned tooth clockwise or counter‐clockwise, calculating OPC at each new orientation, and averaging all the OPC values together.^60^ Orientation is similar to OPC, but the data do not need to be binned.^43^, ^55^ Because of the mathematical similarities in these metrics, all conclusions drawn from orientation/OPC/OPCR discussed herein should be considered interchangeable, although the exact values are not interchangeable.

In primates, OPC is a poorer indicator of diet, showing large levels of overlap between species with distinctly different diets,^6^, ^42^, ^43^, ^45^, ^47^ possibly because there is a lower level of variation in dental complexity within primates compared to other mammalian clades. In contrast, correlations between OPC and diet are present at higher taxonomic levels. For example, herbivores had higher OPC than omnivores in carnivores and rodents, but the opposite is true in bats and platyrrhines.^6^, ^50^, ^61^

### 2.6 Relief index and occlusal relief

Relief index (RFI) and occlusal relief (OR) are mathematically identical, taking the ratio of tooth surface area to cross‐sectional area (a proxy for size). They differ in that RFI takes into account the entire enamel cap (EEC), while OR takes into account only the portions of the tooth superior to the lowest point on the occlusal surface (basin cutoff, BCO; see Box 3).^10^, ^62^, ^63^ Relatively taller crowned teeth have more surface area for their size and higher RFI. In this respect, RFI can act as a hypsodonty index—teeth that are hypsodont have higher RFI than those that are brachydont. Teeth with relatively tall cusps have high OR. Therefore, RFI can measure “crown hypsodonty” and OR “cusp hypsodonty.” Primates with taller crowned/cusped molars, like folivores and insectivores, have higher RFI/OR than those with lower crowned/cusped molars, like frugivores and hard‐object feeders.

### 2.7 Shearing crest length

The term “shearing crest length” is somewhat ambiguous and can be used to describe the SQ and SR. Here, it is used to describe a specific, landmark free method used to quantify the length of both primary and secondary (compensatory) shearing crests in some primate studies. Like OPC, this method first determines the normal direction of each triangle, but only uses two bins: east and west (i.e., buccal and lingual). A transition from buccal to lingual facing triangles indicates a peak and thus the presence of a crest. The sum of the length of the peaks quantifies shearing crest length.^27^, ^47^ This metric will likely yield similar results as SQ, SR, and other metrics that quantify shearing capability, but has the advantage of being able to be calculated on worn teeth.

### 2.8 Slope and inclination

Slope is the derivative of, or change in, elevation over the surface of the tooth.^40^ Inclination is similar to slope, but measured differently. Assuming a tooth is oriented/aligned during scanning so the occlusal surface is pointed in the +z direction, inclination is the angle between the vector normal to the triangle in the −*z* direction and the horizontal, *xy* plane.^43^ Slope and inclination are not measures of sharpness, and relate to diet in the same manner as angularity and curvature. Teeth with taller cusps will have steeper slopes/inclinations. As such, slope/inclination values appear to relate to diet similarly to RFI/OR, but have not been extensively used in dietary reconstructions.^43^, ^45^

## 3 DENTAL TOPOGRAPHY METRICS

### 3.1 Averages, sums, or distributions?

Topographic metrics are usually measured at several locations over the tooth's surface and averaged (e.g., PCV, angularity) or summed (e.g., OPC, DNE): only two metrics (RFI/OR and shearing crest length) produce one measurement per tooth. Averaged/summed metrics provide information concerning whole tooth shape, and location specific information can be useful when analyzing location specific questions about shape. For example, the correlations between location specific values for elevation, inclination, orientation, and curvature on the enamel–dentine junction (EDJ) and outer enamel surface (OES)^55^ were calculated to investigate the influence of EDJ shape on OES shape. It can also be used to address questions about certain portions of the tooth (e.g., shape of the mesial vs. distal half).^64^

### 3.2 Comparability of topographic metrics

Several of the topographic metrics are conceptually/geometrically similar and compute similar aspects of dental form. For example, DNE,^41^ angularity,^40^ and curvature^43^ all measure tooth curviness/sharpness, but differences in the mathematics behind these metrics mean that values cannot be interchanged, with the correlation between variable being potentially extremely weak (e.g., in platyrrhines, DNE and angularity are weakly linearly correlated, *p* = .018, *r* ^2^ = 0.043).^56^ While several methods exist for measuring the same aspect of dental morphology, it is difficult to pick the “best” metric for quantifying a distinct aspect of dental morphology, as the relationship between dental shape quantified through dental topography and diet can vary between clades.^6^ For example, DNE is effective at differentiating molars of folivorous from frugivorous platyrrhines,^6^ but angularity is not.^45^ Conversely, DNE is ineffective at predicting diet in hominoids—unless sympatric species are being compared, as character displacement has occurred in hominoid diet and tooth morphology^54^—but angularity is potentially effective.^40^, ^47^ It is further difficult to pick the “best” metric as no studies use all metrics, and not all studies use the same molar, making it difficult to compare results across studies.

Dental topographic metrics that quantify conceptually/geometrically dissimilar aspects of dental form are also often correlated, but the strength and significance of the correlations vary.^41^, ^42^, ^43^, ^44^, ^45^, ^65^ The presence and strength of such correlations could be affected by parameters such as dietary variability encompassed by the sample, degree of phylogenetic relatedness, and method/resolution of data acquisition. For example, the relationship between RFI and DNE is strong in prosimians (*R* ^2^ = 0.736)^41^ but not South African hominins (*R* ^2^ = 0.254–0.428, depending on the method used for DNE).^42^

Despite these and other problems, some mathematical relationships exist, making the following generalities possible.

1. *Average slope/inclination and OR are strongly correlated*. For a given cross‐sectional area, teeth with increased surface area will be relatively taller, and cusps will require steeper slopes to reach the bottom of the basins.
2. *Orientation, OPCR, and OPC are correlated, but values are not interchangeable*.
3. *DNE, angularity, and curvature may be correlated in some situations, but highly uncorrelated in others*.^43^, ^45^, ^56^
4. *RFI and OR can be completely uncorrelated, with RFI quantifying relative crown height and OR relative cusp height*.
5. *PCV and RFI/OR are correlated*. Relatively taller crowned/cusped teeth hide the sides of the tooth/cusps and basins from ambient light more effectively than relatively lower crowned/cusped teeth, making PCV and RFI/OR correlated, but the two metrics can produce differing results (e.g., *A. africanus* and *P. robustus* differed in RFI, but not PCV^42^).

Through all studies, a general consensus has developed between primate tooth shape and diet: primates that require a high chewing efficiency tend to have sharper, more complex, higher‐crowned, and morphologically wear‐resistant molars.

### 3.3 Effects of wear and age

Being a landmark free method, dental topography is often used to investigate the effects of wear on tooth shape^40^, ^42^, ^47^, ^63^, ^65^, ^66^, ^67^, ^68^; when created, this was one of the stated advantages of dental topography.^40^ Dental wear changes tooth shape, but the magnitude and direction of that change depends on the taxa and metric. As molars wear, wear facets begin to form, potentially altering complexity and curvature. Cusps begin to decrease in height, becoming flatter/rounder, and eventually dentine becomes exposed, producing an enamel ridge around the dentin pool that acts as a compensatory crest. Dentin pools increase in size and the enamel ridge increases in length with age up until a point, when the dentin pools converge and there is a drastic decrease in enamel ridge length. In *Propithecus edwardsi*, this corresponds with a decrease in chewing efficiency and infant survival rate.^27^

Dental topography can be used to analyze assemblages/collections of worn teeth, but teeth of different wear stages cannot be directly compared. Table 1 in a study by Glowacka and colleagues^47^ summarized the relationship between dental wear and topographic metrics in studies published prior to 2016. In general, molars either maintain or lose sharpness, complexity, and relative height with wear. Table 2 and S3‐S5 in a study by Berthaume and colleagues^42^ showed that morphological wear resistance (PCV) increases with wear, and King and colleagues^27^ showed that shearing crest length can increase with age. The variable relationship between wear and topographic metrics prevents teeth from being indiscriminately compared. Instead, level of wear^40^, ^42^, ^63^, ^66^, ^69^ or actual^27^, ^47^, ^70^/estimated^65^, ^68^ age should be held constant.

Dental topographic methods have been used to investigate how wear could be potentially adaptive. In the future, these data can be used to (a) investigate adaptive tradeoffs between dental form and musculoskeletal/digestive systems (e.g., mandibular morphology) in response to dietary mechanical properties,^71^, ^72^ (b) illuminate how teeth are adapted to nondietary aspects of the environment (e.g., dust/grit load),^42^ and (c) be used to generate hypotheses about why some species wear their teeth quicker than others.

### 3.4 Sensitivity to data acquisition and processing

Most topographic metrics are sensitive to data acquisition and processing^41^, ^42^, ^49^, ^52^, ^56^, ^73^, ^74^ (Box 3). Due to time constraints, sensitivity studies generally investigate the effect of one or two parameters (e.g., triangle count,^42^, ^73^ smoothing,^10^, ^49^ cropping^41^, ^75^) on one tooth. If the topographic metric changes minimally, the effect of the parameter is considered negligible. Unfortunately, this approach suffers from small sample sizes and does not investigate the effect of these parameters on the relationship between dental topography and diet. A study is currently in review investigating the effects of triangle count, resolution, smoothing, and cropping on the correlative and predictive effects of DNE, OPCR, RFI, and PCV.^52^

Summative metrics and metrics that analyze triangles in (near) isolation, such as DNE and OPCR, are sensitive to triangle count and smoothing.^33^, ^42^, ^54^, ^76^ At high triangle counts, both RFI and OR are relatively insensitive to triangle count and smoothing.^10^, ^73^ Average angularity, curvature, and shearing crest length are likely sensitive to smoothing, as smoothing erases sharp edges, and average slope and inclination are likely less affected, as smoothing will not decrease tooth height. One newly introduced metric, ariaDNE, has the ability to robustly quantify surface curviness, and appears insensitive to all processing assumptions, except for cropping.^58^ All metrics will be affected by cropping, as cropping changes the shape of the surface being analyzed.

### 3.5 What metrics should be used?

Not all metrics are appropriate for all studies. If dental variation in a small group of closely related primates is being compared, OPCR is often not informative due to low variation in dental complexity.^6^, ^42^ PCV, DNE, angularity, curvature, slope, inclination, RFI, and OR would be more appropriate, given their ability to pick up subtle, subspecies, and population level differences in diet.^40^, ^54^, ^63^, ^66^, ^70^, ^77^

When quantifying tooth shape, studies tend to use several metrics, together. If only one metric is used, it is possible the aspects of dental morphology that vary between taxa are not being quantified, and it may lead authors to conclude taxa have similar dietary ecologies, when they do not. Using multiple metrics increases confidence in results by accounting for numerous aspects of tooth shape. We recommended using at least four topographic metrics (for sharpness, complexity, relative tooth/crown height, and morphological wear resistance), in conjunction with tooth size (as it increases the predictive power of dental topography),^6^, ^41^, ^62^ as there are some aspects of dietary ecology captured by tooth size and not tooth shape (e.g., maximum bite force). This framework was used to reconstruct the diet of *H. naledi*: similarities in DNE and OPCR implied that *H. naledi*'s diet had similar fracture properties to the other hominins, but differences in RFI, PCV, and tooth size implied that its diet was more abrasive.^42^

## 4 DENTAL TOPOGRAPHY AND EVOLUTION

### 4.1 Natural selection, dental topography, and diet

From a dental perspective, mastication is a biomechanical process where foods are trapped/stabilized, broken down, and cleared away, all while teeth resist permanent damage.^78^ Natural selection is likely acting on tooth shape through one or more of these functions, and the relative importance of these functions depends on diet. For example, trapping and stabilizing foods (herein trapability)^78^ is likely more important for animals with diets requiring high bite forces as they need to transfer large forces to the food without it slipping, while food breakdown efficiency is more important for diets consisting of foods difficult to digest.

The first publications on dental topography suggested basin volume and drainage could be used to quantify trapability and food clearance, but these metrics were later dismissed.^37^, ^39^ No subsequent topographic metrics have quantified trapability or food clearance, and it is therefore unknown how these factors relate to dental function and diet in primates.

The majority of aspects of dental morphology related to longevity (tooth size, enamel thickness, enamel microstructure, and fracture risk)^79^, ^80^ are related to internal dental structure/geometry and not quantified by dental topography. As PCV can quantify morphological wear resistance, it could potentially be used to quantify morphological dental longevity. Another metric, RFI, may also be able to predict the maximum lifetime, and therefore longevity, of a tooth, as it quantifies relative tooth height. While primates with abrasive diets have increased relief and morphological wear resistance (e.g., folivores), primates with nonabrasive diets can have higher and lower relief and morphological wear resistance (e.g., insectivores and frugivores), making it possible, but unlikely, that selection is acting on tooth shape to increase morphological longevity.^6^, ^51^

Selection is likely working on other topographic metrics through food breakdown. Tooth shape is correlated to chewing efficiency,^4^, ^17^, ^19^ which is positively correlated to both digestive efficiency and caloric intake^19^, ^27^, ^81^, ^82^: this provides an evolutionary pathway through which selection can act on tooth shape, and thereby dental topography, in animals that require high chewing efficiencies (i.e., insectivores and folivores).^4^ For primates with relatively lower chewing efficiencies (e.g., frugivores, hard‐object eaters), selection is not acting strongly in favor of chewing efficiency, and selection is likely acting strongly on an aspect of food breakdown independent of chewing efficiency.

What is being selected for in these groups? Researchers have suggested frugivores need to juice foods, and the most effective way to do this is through dull cusps and large basins (i.e., the mortar and pestle hypothesis).^9^, ^15^, ^83^, ^84^ However, no experiments have compared the benefits of juicing foods versus cutting foods into small enough pieces to be swallowed, and how this would result in an increased evolutionary fitness.

A range of hypotheses exist governing the relationship between cusp/tooth shape and hard‐object feeding. For complete descriptions of these hypotheses, and references supporting their formation, please see papers by Berthaume and colleagues.^85^, ^86^ Briefly, the Blunt Cusp Hypothesis comes from comparative anatomy and predicts dull cusps are better for hard‐object feeding, potentially because they reduce masticatory force and/or energy.^11^, ^26^, ^67^, ^85^ The Strong Cusp Hypothesis comes from contact mechanics, and similarly predicts dull cusps are better for hard‐object feeding, but because it reduces enamel stresses, decreasing risk of enamel fracture. Conversely, the Pointed Cusp Hypothesis, also from contact mechanics, predicts sharp cusps are better because they increase stresses in the food item.^85^, ^87^, ^88^, ^89^ Cusp sharpness is certainly correlated for food item breakdown in single cusped teeth^87^, ^88^, ^89^ and symmetrical molars,^86^ but physical experimentations and finite element models failed to find support for these hypotheses in multicusped, asymmetrical molars.^85^, ^86^, ^90^

From these studies, the Complex Cusps Hypothesis emerged, which states hard‐object feeders should maximize the stresses in the food item while minimizing stresses in the enamel. As a result, multicusped, asymmetrical teeth should have a combination of sharp and dull cusps where one dull cusp transfers the majority of forces to the food item while the others act to stabilize the food, promoting food item failure while preventing enamel fracture. Looking at the ratio of stresses in the food item to stresses in the enamel, a hemispherical food item and a set of four cusped hypothetical molars, the authors found support for this hypothesis^86^ across a range of food item sizes.^90^ A later study tested the relationship between dental topography and energy, stresses in the food, stresses in the enamel, and the ratio of these stresses using the hypothetical molars, but found no relationship between shape and function.^44^ The mechanical reason why hard‐object feeding primates tend to have low crowned, bulbous molars remains unknown, possibly because (a) natural selection is acting on tooth shape in a way not encompassed by those hypotheses or experiments, or (b) selection is not acting on tooth shape at all in hard‐object feeders, but another factor (e.g., enamel thickness)^55^ that covaries with tooth shape (e.g., see *Biological sources of variation in tooth shape*).

Much more research is needed to unveil the complex relationship between tooth shape and function in primates, particularly to understand how selection is working on molar shape in frugivores and hard‐object feeders.

### 4.2 Heritability

Despite understanding the heritability of some aspects of dental morphology,^91^, ^92^ we have no understanding of the heritability of biomechanically relevant aspects of molar occlusal morphology and how it relates to EDJ shape and/or enamel secretion patterns in primates.^93^ This is necessary to construct evolutionary models to (a) understand how selection is acting on dental topography and (b) perform more accurate dietary reconstructions, by understanding how long it takes teeth to become adapted to diet. Here, the biggest challenge lies in gaining a pedigreed collection of unworn dental molds: worn teeth cannot be used for these purposes, as their shape is a product of genetic and environmental factors.^94^

### 4.3 Developmental sources of variation in occlusal topography

Unlike bone, dental enamel does not remodel, meaning changes in unworn occlusal topography occur because of changes in dental growth and development. During growth and dental development, enamel is deposited by ameloblasts traveling from the EDJ toward the OES,^79^ making the shapes of the EDJ and OES correlated.^48^, ^92^, ^95^ Therefore, it is possible that variation in EDJ shape and/or enamel deposition may be responsible for the variation in occlusal topography.

Three studies investigated the relationship between dental growth and development and dental topography. The first study discovered the following three relationships between EDJ and OES complexity (a) OPC in the EDJ and OES are similar, (b) OES OPC is moderately higher than EDJ OPC, and (c) OES OPC is much higher than EDJ OPC.^95^ Skinner and colleagues^95^ concluded that OES complexity is controlled primarily by the EDJ in first and second relationships, but enamel deposition in third relationship, and EDJ complexity can provide a lower limit for OES complexity (i.e., OES OPC ≥ EDJ OPC).

The second study investigated relationship between EDJ shape, OES shape, and enamel thickness, and concluded that the inclination, orientation, and curvature of the EDJ and OES were highly correlated, and OES mean curvature was affected by enamel thickness.^55^ The correlation between enamel thickness and OES shape requires further investigation. Finally, the third study combined their results with Guy and colleagues^55^ and found a stronger correlation between EDJ and OES in DNE, RFI, and OPCR within nonprimate Euarchonta compared with primates,^96^ implying that primate OES is determined more by enamel deposition than EDJ morphology. However, Selig and colleagues^96^ directly compared DNE and curvature to come to this conclusion, and as previously stated, these values are not directly comparable.

### 4.4 Dietary mechanical properties

Mechanical properties are the intensive (size independent) properties of a material that describe how the foods behave under a load.^8^ Dietary mechanical properties are the cumulative set of mechanical properties for a diet. They are often measured by following an animal/set of animals in the field, and testing the mechanical properties of the foods they consume.^8^, ^9^ Collection of dietary mechanical properties is challenging, requiring researchers to follow primates in the field, collect foods that are being consumed from the exact site/plant they are being foraged, properly store foods for transport, and test the properties of those foods within 24 hours using a (portable) universal tester. Ideally, foods that the primates are actively consuming, and not those nearby, are tested, as there may be differences in mechanical properties between these foods. In the field, foods must be tested relatively quickly, or their mechanical properties will begin to change.^8^, ^9^

Presumably, different diets have different sets of mechanical properties, and different tooth shapes are better/worse at breaking down foods with different sets of mechanical properties. Generally, plant and animal‐based structural fibers require large amounts of energy to cut, and animals with high‐fiber diets have sharper teeth^41^, ^97^ to cut fibers efficiently. Comparative work in the great apes^54^ provides support for the relationship between tooth sharpness and dietary plant‐based fiber in frugivores and folivores. Comparative work on insectivorous primates^4^ and nonprimate mammals^50^ supports the relationship between tooth sharpness and animal‐based structural fiber (although it is unclear if the results in nonprimate mammals are congruent with primates). Within hominins, an increase in tooth sharpness, as was observed in South African hominins relative to extant great apes,^42^ could indicate a diet higher in plant or animal‐based fiber intake. Combining standard dietary reconstruction methods like dental microwear, isotope analyses, and phytolith identification in dental calculus^98^ which record short‐term (days, years) dietary signatures with methods like dental topography which record long‐term (generations) dietary signatures can provide more comprehensive dietary reconstructions.

Three studies have investigated the relationship between dietary mechanical properties and tooth shape. One study used both the wedge and scissors tests to quantify the energy release rates (i.e., “toughness,” see Berthaume^8^ for the relationship between energy release rate and toughness) for a number of foods consumed by geladas. The wedge/scissors tests estimate the energy release rate by fracturing an item with a wedge/pair of scissors, and dividing the energy needed to fracture by the newly formed surface area. The wedge causes fracture through tensile forces (mode I failure) and the scissors primarily through shear forces (mode III failure), and the results of these two tests are rarely comparable, often producing statistically significantly different results for the same foods (see figure 13 in Berthaume^8^). For example, when the energy release rate, or toughness is measured for ginger using the wedge test, the average energy release rate is 1,907.63 ± 635.03 J/mm^2^. But when measured using the scissors test, the average energy release rate is 666.87 ± 173.44 J/mm.^8^ As data gathered using both methods was not dealt with separately,^81^ any relationship between tooth shape and dietary mechanical properties may be valid. Another study utilizing just the scissors test found a relationship between dietary mechanical properties and dental topography in three populations of *Lemur catta*.^77^ The last study used the scissors test and found a positive correlation between chewing efficiency and tooth size, quantified by both surface area and cross‐sectional area.^82^ More work combining dental topography and dietary mechanical properties is needed.

## 5 WHAT ELSE CAN DENTAL TOPOGRAPHY TELL US?

### 5.1 Fallback foods vs. primary diet

Dental topography was first used to investigate the effects of fallback foods (i.e., foods eaten when preferential foods are unavailable)^99^ on molar shape in *Pan Troglodytes troglodytes* and *Gorilla gorilla gorilla*. Both species have similar primary diets, but dissimilar fallback diets, and differences in molar shape were hypothesized to reflect differences in fallback foods. These conclusions were used to reconstruct hominin fallback foods.^40^, ^63^, ^67^ However, without an outgroup, it is not possible to tell if these differences reflect dietary differences or phylogenetic history. A subsequent study using the same metrics showed dental topography reflects both primary and fallback foods in platyrrhines.^45^

A study on great apes showed DNE reflects a) primary diet when sympatric species are compared, and b) differences in dietary fiber.^54^ Based on these results, it was suggested South African hominins *A. africanus*, *P. robustus*, and *H. naledi* may have had diets higher structural fiber than the great apes, but it was not possible to tell if the structural fiber came from a plant or animal source,^42^ and if a plant source, whether it is coming from above ground or underground storage organs.^100^

The primary barrier in investigating the relationship between tooth shape and primary and fallback foods comes from the classification of fallback foods. Fallback foods are “items assumed to be of relatively poor nutritional quality and high abundance, eaten particularly during periods when preferred foods are scarce (p. 1220 in Marshall and Wrangham^99^).” Using this definition, items, such as aquatic and terrestrial herbaceous vegetation (AHV, THV), are classified as fallback foods.^101^ However, AHV and THV are sometimes preferentially consumed by *G. g. gorilla* when fruits are readily available,^102^ suggesting, in these situations, they are not fallback foods, but preferred ones. The same is true for *Homo sapiens* today, which sometimes pass over what would be classified as “preferred foods” (e.g., meat, fruits) for what would be classified as “fallback foods” (e.g., leafy green vegetables). To understand the relationship between fallback foods and dental form, a definition is first needed that does not classify preferred foods as fallback ones.

### 5.2 Non‐dietary applications of dental topography

Dental topographic studies focus on diet, but the method can be used for more. Eronen and colleagues^35^ used dental topography to investigate the long‐term effects of climate change on primate conservation. Shifts in weather patterns and rainfall are causing the greater bamboo lemur (*Prolemus simus*) to spend more time eating mature, mechanically challenging bamboo, which wears its teeth faster. Using the paleontological record, they showed that when similar shifts happened elsewhere in Madagascar, localized extinction of bamboo specialists occurred.^33^ Godfrey and colleagues^97^ used dental topography to investigate long‐term ecological changes in primates in Madagascar, showing how the giant extinct lemurs occupied ecological niches currently unoccupied by extant lemurs, and how their extinction changed the ecology of the extant lemurs.^97^

The effects of long‐term interspecific competition can be difficult to quantify. Using dental topography, Berthaume and Schroer^54^ showed how indirect, intertaxon dietary competition led to character displacement in African great ape molar shape. They hypothesized this framework could be used to investigate dietary competition in extinct hominins, and that competition between *Paranthropus* and early *Homo* may have led to the evolution of each clade.^54^ Similarly, Boyer and colleagues^103^ observed differences in plesiadapid dental topographic metrics, and suggested competition between a Paleocene population of *Plesiadapis cookie* and *P. tricuspidens* may have led to character displacement and the eventual evolution of *Platychoerops*. Prufrock and colleagues^74^ also used dental topography to investigate plesiadapid evolution and found evidence of dietary competition between *Chiromyoides* and rodents. Finally, Boyer and colleagues^103^ used dental topography to quantify tooth shape in early primates, and based on differences, identified a new species.

## 6 THE NEXT 20 YEARS

### 6.1 Ground‐truthing

The largest barrier facing dental topographic studies is the lack of a relationship between dental form and masticatory performance. The first studies to investigate the relationship between dental form and masticatory performance by Kay and Sheine found a tooth's shearing capability was an efficient predictor of chewing efficiency in two primate, and one non‐primate, mammal species.^4^, ^17^, ^19^ One more recent study investigated the relationship between four dental topographic metrics and biomechanics using a computational modeling approach. Berthaume^44^ constructed a parametric model of a four cusped molar and used finite element analysis (FEA) to investigate the relationship between DNE, OPCR, RFI, and PCV and stresses in the food item, stresses in the enamel, the ratio of these two metrics, and energy absorbed by the food item during hard food item biting. However, no correlation was found between the dental topographic and functional parameters. Laird and colleagues^82^ investigated the relationship between chewing efficiency, one dental topographic metric (slope), and metrics for tooth size in modern humans using an in vivo experimental set up. They found chewing efficiency was not correlated to slope, but was positively correlated to tooth size, indicating larger teeth chewing more efficiently.

Barring these studies, little has been done to investigate the relationship between these dental topographic metrics and masticatory performance, begging the question: all else being equal, *do dental topographic metrics actually correlate to food breakdown during mastication?* This question goes beyond dental topography, and cuts to the heart of dental functional morphology. For this field to move forward efficiently, we require a ground‐truth relationship between these shape metrics and masticatory performance.

Some additional issues that are often ignored must also be addressed for the field to move forward and are discussed briefly later.

### 6.2 Standardization of metrics

One of the challenges of dental topography is the numerous methodologies for quantifying tooth shape. New metrics may not be needed, unless they can quantify other aspects of dental form currently being ignored, or aspects of dental form directly related to masticatory performance. An increased understanding of metric comparability, particularly of metrics that quantify similar aspects of dental form, is needed for study comparability.^56^ Ideally, a standardized methodology for performing analyses, complete with a standardized set of metrics that are functionally significant, will also be developed and adapted.

### 6.3 Scale

The issue of scale may be relevant both in terms of animal size and the scale of the question being asked. The selective pressures acting on tooth shape may be stronger in small primates than large ones, as large primates can compensate for ineffective tooth shape with absolutely larger muscles and bite forces. Small‐scale evolutionary questions, such as dental adaptations in two populations of the same species with distinct diets, may be difficult/not possible to address with dental topography, as changes in dental form over the time the two species have been isolated may be too small to be quantified through dental topography. Dental topographic studies have shown dietary signals can be obtained from hominoid molars:^40^, ^42^, ^54^, ^63^, ^67^ this suggests that, even in species with relatively long life histories, dietarily meaningful changes in molar topography can accumulate in hundreds of thousands of years.

### 6.4 Population level variation

Similarly, little is known about population level variation in dental topography. One study showed population level differences in *Lemur catta*,^77^ and another on atelids showed population differences in tooth wear, but not shape.^104^ Population level studies, especially those that include genetic, genomic, and/or proteomic data, will help explain how quickly diet can act on tooth shape through natural selection and provide valuable insights into the possible effects of gene flow, genetic drift, and other evolutionary mechanisms on tooth shape. This will further aid clarifying the use of dental topographic metrics in detecting new species in the fossil record.

### 6.5 Sexual dimorphism

Sexually dimorphic differences in dental characters sometimes exist independent of size.^105^ In dental topographic studies, sexual dimorphism is often ignored, and differences between species are assumed to be greater than differences between sexes. This may or may not a valid assumption, particularly when considering primates with large levels of body mass sexual dimorphism, such as *Theropithecus*, *Pongo*, and *Gorilla*, and there is evidence to suggest primates with large levels of body mass sexual dimorphism have dimorphic diets.^106^

### 6.6 Does body mass matter?

Small primates are more limited in their ability to forage over long distances and produce high bite forces, meaning they need to be more efficient to survive. Larger primates have the luxury of being less efficient, as they may already possess tools that are “good enough” for their function due to allometry. The shorter intergenerational times of smaller primates also implies the cumulative effects of selection acting on tooth shape may become apparent over a shorter period of time, potentially making the correlation between tooth shape and diet stronger in smaller primates.

Since dental topography quantifies shape, it should be independent of tooth size, implying topographic metrics do not need to be normalized by size. This is supported by dental topographic studies which find a correlation between tooth shape and diet across a broad range of body sizes.^6^, ^10^, ^12^, ^45^, ^51^ But larger teeth have the potential to hold more features, and more triangles may be needed to capture their shape digitally.^54^, ^97^ Together, this means size may be important to dental topographic studies for both biological and methodological reasons.

### 6.7 What role does grit and dust play in molar shape?

Both RFI and PCV are well suited to investigate the effects of environment on molar shape. It is possible teeth with higher RFI are better adapted to more abrasive diets, and if other topographical parameters, such as DNE and OPCR, are constant, differences in RFI may reflect differences in grit/dust consumption.^42^ Similarly, as PCV measures morphological wear resistance, it may also be useful in investigating environmental factors, such as grit/dust, related to dental wear.

### 6.8 Are crenulations important?

Most studies investigating tooth sharpness simplify teeth to the point where crenulations begin to disappear^5^, ^6^, ^54^ (c.f.^55^). However, crenulations have biomechanical consequences, as a smooth surface will transmit forces to an object differently from a “bumpy” surface. In primates, they are hypothesized to “grip” foods,^7^ which is why they are believed to be present in hard‐object feeders. Functionally, it is possible that crenulations could also cut fibers: after all, crenulations increase tooth sharpness and complexity.^6^, ^54^ If crenulations do act as a cutting surface, they play an important, unrecognized biomechanical function that should be considered in dental topographic analyses. This could explain how species with low SQ and crenulated cusps could be efficient folivores.^107^, ^108^

The absence of crenulations from the most highly folivorous primates, for which cutting is important, could challenge the hypothesis that crenulations are acting as a cutting surface. However, these species generally possess molars with high OR, and it is possible either crenulations or high OR, and not both, are needed to create an efficient cutting surface. The degree of molar crenulation will also likely be important in testing this hypothesis, as it is possible that crenulated molars do not become efficient at cutting until a certain degree of crenulations is reached. Biomechanical studies are needed to address this question.

### 6.9 Does molar shape matter in modern humans?

After the advent of stone tools, cooking may have greatly relaxed the selective pressures working on tooth shape in modern humans. (Note: in Berthaume and colleagues' study,^42^ the lack of lithics or evidence of controlled fire use for *H. naledi* led the authors to hypothesize that selection was still acting on tooth shape in *H. naledi* the same way it was in other primates.) However, dental morphology may still reflect diet in certain situations. For example, the advent of agriculture led to an increase in carbohydrate consumption and dental caries. It is possible that more complex teeth have more places for cavity‐causing bacteria to hide, and therefore selection may have acted against complex teeth. To date, no studies have investigated modern human variation in dental topography.

## 7 CONCLUDING REMARKS

The amount we have learned about primate teeth and function is astounding. We have a better idea of how tooth shape relates to diet than ever before. But, at the same time, the question of why the variation in primate molars exists is far from being answered. Diet is a major factor in determining molar shape, but many mysteries still surround the evolutionary pathways that relate tooth shape and diet. In some clades, chewing efficiency and energy are important, while in others these factors matter less.

The complex relationship between dental development, molar shape, and how EDJ shape and ameloblasts affect dental function is only beginning to be understood. Other questions require much more experimental/simulated data which, together, can address some of the big questions surrounding primate evolution. With time, dental topography could be used to predict future trend in extant primate evolution. And in the hand of conservationists, these data could help predict the extinction risk of some primates and help establish protocols to prevent their demise.^33^

What an exciting time it is to be studying primate dental topography!

## Data Availability

Data availability statement is not applicable for this study as no original data has been used.

## References

[1] Ungar PS . 2017 Evolution's bite: a story of teeth, diet, and human origins, Princeton, NJ: Princeton University Press.

[2] Hiiemae KM . 1967 Masticatory function in mammals. J Dent Res 46:883–893. 5234390 10.1177/00220345670460054601

[3] Hiiemae KM . 2000 Feeding in mammals In: Schwenk K, editor. Feeding: form, function, and evolution in tetrapod vertebrates, San Diego: Academic Press p 411–448.

[4] Kay RF , Sheine WS . 1979 On the relationship between chitin particle size and digestibility in the primate *Galago senegalensis* . Am J Phys Anthropol 50:301–308.

[5] Ledogar JA , Winchester JM , St. Clair EM , Boyer DM . 2013 Diet and dental topography in pitheciine seed predators. Am J Phys Anthropol 150:107–121. 23212472 10.1002/ajpa.22181

[6] Winchester JM , Boyer DM , St. Clair EM , Gosselin‐Ildari AD , Cooke SB , Ledogar JA . 2014 Dental topography of platyrrhines and prosimians: Convergence and contrasts. Am J Phys Anthropol 153:29–44. 24318939 10.1002/ajpa.22398

[7] Kinzey WG . 1992 Dietary and dental adaptations in the Pitheciinae. Am J Phys Anthropol 88:499–514. 1503121 10.1002/ajpa.1330880406

[8] Berthaume MA . 2016 Food mechanical properties and dietary ecology. Am J Phys Anthropol 159:79–104. 10.1002/ajpa.22903 26808100

[9] Lucas PW . 2004 Dental functional morphology: how teeth work, Cambridge: Cambridge University Press.

[10] Boyer DM . 2008 Relief index of second mandibular molars is a correlate of diet among prosimian primates and other euarchontan mammals. J Hum Evol 55:1118–1137. 18930306 10.1016/j.jhevol.2008.08.002

[11] Luke DA , Lucas PW . 1983 The significance of cusps. J Oral Rehabil 10:197–206. 6575161 10.1111/j.1365-2842.1983.tb00113.x

[12] Thiery G , Gillet G , Lazzari V , Merceron G , Guy F . 2017 Was Mesopithecus a seed eating colobine? Assessment of cracking, grinding and shearing ability using dental topography. J Hum Evol 112:79–92. 29037418 10.1016/j.jhevol.2017.09.002

[13] Hylander W . 1975 Incisor size and diet in anthropoids with special reference to Cercopithecidae. Science 189:1095–1098. 808855 10.1126/science.808855

[14] Ungar PS . 1996 Relationship of incisor size to diet and anterior tooth use in sympatric sumatran anthropoids. Am J Primatol 38:145–156. 31918473 10.1002/(SICI)1098-2345(1996)38:2<145::AID-AJP3>3.0.CO;2-Z

[15] Lucas PW , Luke DA . 1984 Chewing it over: Basic principles of food breakdown In: Chivers DJ, Wood BA, Bilsborough A, editors. Food Acquisition and Processing in Primates, Boston, MA: Springer, US p 283–301.

[16] Helkimo E , Carlsson GE , Helkimo M . 1978 Chewing efficiency and state of dentition. Acta Odontol Scand 36(1):33–41. 273364 10.3109/00016357809026364

[17] Sheine WS , Kay RR . 1982 A model for comparison of masticatory effectiveness in primates. J Morphol 172:139–149. 7097769 10.1002/jmor.1051720202

[18] Fritz J , Hummel J , Kienzle E , Arnold C , Nunn C , Clauss M . 2009 Comparative chewing efficiency in mammalian herbivores. Oikos 118:1623–1632. 10.1016/j.cbpa.2009.07.016 19651229

[19] Sheine WS , Kay RF . 1977 An analysis of chewed food particle size and its relationship to molar structure in the primates *Cheirogaleus medius* and *Galago senegalensis* and the insectivoran *Tupaia glis* . Am J Phys Anthropol 47:15–20.

[20] Kay RF . 1975 The functional adaptations of primate molar teeth. Am J Phys Anthropol 43:195–216. 810034 10.1002/ajpa.1330430207

[21] Anthony MRL , Kay RF . 1993 Tooth form and diet in ateline and alouattine primates: Reflections on the comparative method. Am J Sci 293:356–382.

[22] Boyer DM , Winchester J , Kay RF . 2015 The effect of differences in methodology among some recent applications of shearing quotients. Am J Phys Anthropol 156:166–178. 25256698 10.1002/ajpa.22619

[23] Strait SG . 1993 Differences in occlusal morphology and molar size in frugivores and faunivores. J Hum Evol 25:471–484.

[24] Strait SG . 1993 Molar morphology and food texture among small‐bodied insectivorous mammals. J Mammal 74:391–402.

[25] Ungar PS , Kay RF . 1995 The dietary adaptations of European Miocene catarrhines. Proc Natl Acad Sci U S A 92:5479–5481. 7777533 10.1073/pnas.92.12.5479 PMC41718

[26] Kay RF . 1981 The nut‐crackers—A new theory of the adaptations of the Ramapithecinae. Am J Phys Anthropol 55:141–151.

[27] King SJ , Arrigo‐Nelson SJ , Pochron ST , et al. 2005 Dental senescence in a long‐lived primate links infant survival to rainfall. Proc Natl Acad Sci U S A 102:16579–16583. 16260727 10.1073/pnas.0508377102 PMC1283847

[28] Kay RF , Hylander WL . 1978 The dental structure of mammalian folivores with special reference to primates and Phalangeroidea (Marsupialia) In: Montgomery GG, editor. The Ecology of Arboreal Folivores, Washington, D.C.: Smithsonian Institution Press p 173–191.

[29] Lucas P , Constantino P , Wood B , Lawn B . 2008 Dental enamel as a dietary indicator in mammals. Bioessays 30:374–385. 18348196 10.1002/bies.20729

[30] Constantino PJ , Markham K , Lucas PW . 2012 Tooth chipping as a tool to reconstruct diets of great apes (Pongo, Gorilla, Pan). Int J Primatol 33:661–672.

[31] Martin L . 2003 Enamel thickness and microstructure in pitheciin primates, with comments on dietary adaptations of the middle Miocene hominoid Kenyapithecus. J Hum Evol 45:351–367. 14624746 10.1016/j.jhevol.2003.08.005

[32] Dumont ER . 1995 Enamel thickness and dietary adaptation among extant primates and chiropterans. J Mammal 76:1127–1136.

[33] Eronen JT , Zohdy S , Evans AR , et al. 2017 Feeding ecology and morphology make a bamboo specialist vulnerable to climate change. Curr Biol 27(21):3384.e2–3389.e2. 29107552 10.1016/j.cub.2017.09.050

[34] Yamashita N . 2008 Food physical properties and their relationship to morphology: The curious case of kily In: Vinyard C et al., editors. Primate Craniofacial Funct. Biol, New York, NY: Kluwer Academic Press p 387–446.

[35] Cuozzo FP , Sauther ML . 2006 Severe wear and tooth loss in wild ring‐tailed lemurs (*Lemur catta*): A function of feeding ecology, dental structure, and individual life history. J Hum Evol Academic Press. 51:490–505. 10.1016/j.jhevol.2006.07.001 16962643

[36] Cuozzo FP , Sauther ML . 2012 What is dental ecology? Am J Phys Anthropol 148:163–170. 22610892 10.1002/ajpa.21656

[37] Zuccotti LF , Williamson MD , Limp WF , Ungar PS . 1998 Technical note: Modeling primate occlusal topography using geographic information systems technology. Am J Phys Anthropol 107:137–142. 9740307 10.1002/(SICI)1096-8644(199809)107:1<137::AID-AJPA11>3.0.CO;2-1

[38] Jernvall J , Selänne L . 1999 Laser confocal microscopy and geographic information systems in the study of dental morphology. Palaeontol Electron 2:18–905.

[39] Ungar PS , Williamson MD . 2000 Exploring the effects of tooth wear on functional morphology: A preliminary study using dental topographic analysis. Palaeontol Electron 3:1–18.

[40] Ungar PS , M'Kirera F . 2003 A solution to the worn tooth conundrum in primate functional anatomy. Proc Natl Acad Sci U S A 100:3874–3877. 12634426 10.1073/pnas.0637016100 PMC153015

[41] Bunn JM , Boyer DM , Lipman Y , St. Clair EM , Jernvall J , Daubechies I . 2011 Comparing Dirichlet normal surface energy of tooth crowns, a new technique of molar shape quantification for dietary inference, with previous methods in isolation and in combination. Am J Phys Anthropol 145:247–261. 21469070 10.1002/ajpa.21489

[42] Berthaume MA , Delezene LK , Kupczik K . 2018 Dental topography and the diet of Homo naledi. J Hum Evol 118:14–26. 29606200 10.1016/j.jhevol.2018.02.006

[43] Guy F , Gouvard F , Boistel R , Euriat A , Lazzari V . 2013 Prospective in (primate) dental analysis through tooth 3D topographical quantification. PLoS One 8:e66142. 23826088 10.1371/journal.pone.0066142 PMC3691165

[44] Berthaume MA . 2016 On the relationship between tooth shape and masticatory efficiency: A finite element study. Anat Rec 299:679–687. 10.1002/ar.23328 26910570

[45] Ungar PS et al. 2016 Dental topography and diets of platyrrhine primates. Hist Biol 30:1–12.

[46] Winchester JM . 2016 MorphoTester: An open source application for morphological topographic analysis. PLoS One 11:e0147649. 26839960 10.1371/journal.pone.0147649 PMC4739702

[47] Glowacka H , McFarlin SC , Catlett KK , et al. 2016 Age‐related changes in molar topography and shearing crest length in a wild population of mountain gorillas from volcanoes National Park, Rwanda. Am J Phys Anthropol 160:3–15. 26853974 10.1002/ajpa.22943

[48] Tiphaine C , Yaowalak C , Cyril C , et al. 2013 Correlated changes in occlusal pattern and diet in stem murinae during the onset of the radiation of old world rats and mice. Evolution 67(11):3323–3338. 24152010 10.1111/evo.12172

[49] Spradley JP , Pampush JD , Morse PE , Kay RF . 2017 Smooth operator: The effects of different 3D mesh retriangulation protocols on the computation of Dirichlet normal energy. Am J Phys Anthropol 163:94–109. 28218399 10.1002/ajpa.23188

[50] Evans AR et al. 2007 High‐level similarity of dentitions in carnivorans and rodents. Nature 445:78–81. 17167416 10.1038/nature05433

[51] Berthaume MA , Winchester J , Kupczik K . 2019 Ambient occlusion and PCV (portion de ciel visible): A new dental topographic metric and proxy of morphological wear resistance. PLoS One 14:e0215436. 31042728 10.1371/journal.pone.0215436 PMC6493728

[52] Berthaume MA et al. 2019 Effects of cropping, smoothing, triangle count, and mesh resolution on dental topography. PLoS One 14:e0216229. 31059538 10.1371/journal.pone.0216229 PMC6502444

[53] Berthaume MA . 2014 Tooth cusp sharpness as a dietary correlate in great apes. Am J Phys Anthropol 153:226–235. 24227163 10.1002/ajpa.22424

[54] Berthaume MA , Schroer K . 2017 Extant ape dental topography and its implications for reconstructing the emergence of early Homo. J Hum Evol 112:15–29. 29037413 10.1016/j.jhevol.2017.09.001

[55] Guy F , Lazzari V , Gilissen E , Thiery G . 2015 To what extent is primate second molar enamel occlusal morphology shaped by the enamel‐dentine junction? PLoS One 10:e0138802. 26406597 10.1371/journal.pone.0138802 PMC4634312

[56] Pampush JD et al. 2019 Technical note: Comparing dental topography software using platyrrhine molars. Am J Phys Anthropol 169(1):179–185. 10.1002/ajpa.23797. 30768782

[57] Pampush JD et al. 2016 Introducing molaR: A new R package for quantitative topographic analysis of teeth (and other topographic surfaces). J Mamm Evol 23:1–16.

[58] Shan S , Kovalsky SZ , Winchester JM , Boyer DM , Daubechies I . 2019 ariaDNE: A robustly implemented algorithm for Dirichlet energy of the Normal. Methods Ecol Evol 10:541–552.

[59] Evans AR , Jernvall J . 2009 Patterns and constraints in carnivoran and rodent dental complexity and tooth size. J Vertebr Paleontol 29:24A.

[60] Wilson GP et al. 2012 Adaptive radiation of multituberculate mammals before the extinction of dinosaurs. Nature 483:457–460. 22419156 10.1038/nature10880

[61] Santana SE , Strait S , Dumont ER . 2011 The better to eat you with: Functional correlates of tooth structure in bats. Funct Ecol 25:839–847.

[62] Allen KL , Cooke SB , Gonzales LA , Kay RF . 2015 Dietary inference from upper and lower molar morphology in platyrrhine primates. PLoS One 10:e0118732. 25738266 10.1371/journal.pone.0118732 PMC4349698

[63] M'Kirera F , Ungar PS . 2003 Occlusal relief changes with molar wear in *Pan troglodytes troglodytes* and *Gorilla gorilla gorilla* . Am J Primatol 60:31–41. 12784284 10.1002/ajp.10077

[64] Winchester JM . 2016 Molar topographic shape as a system for inferring functional morphology and developmental patterning in extant cercopithecoid primates, Stony Brook University https://search.proquest.com/openview/41acb98c9efda189f1856b1db1f7b6d8/1?pq‐origsite=gscholar&cbl=18750&diss=y

[65] Pampush JD , Spradley JP , Morse PE , et al. 2016 Wear and its effects on dental topography measures in howling monkeys (*Alouatta palliata* ). Am J Phys Anthropol 161:705–721. 27634058 10.1002/ajpa.23077

[66] Klukkert ZS , Teaford MF , Ungar PS . 2012 A dental topographic analysis of chimpanzees. Am J Phys Anthropol 148:276–284. 22610902 10.1002/ajpa.21592

[67] Ungar P . 2004 Dental topography and diets of Australopithecus afarensis and early *Homo* . J Hum Evol 46:605–622. 15120268 10.1016/j.jhevol.2004.03.004

[68] Zohdy S et al. 2014 Teeth, sex, and testosterone: Aging in the world's smallest primate. PLoS One 9:e109528. 25354041 10.1371/journal.pone.0109528 PMC4212904

[69] Bunn JM , Ungar PS . 2009 Dental topography and diets of four old world monkey species. Am J Primatol 71:466–477. 19367586 10.1002/ajp.20676

[70] Dennis JC et al. 2004 Dental topography and molar wear in *Alouatta palliata* from Costa Rica. Am J Phys Anthropol 125:152–161. 15365981 10.1002/ajpa.10379

[71] Swan K. 2016 Dental morphology and mechanical efficiency during development in a hard object feeding primate (*Cercocebus atys*). Ph.D. Thesis University of York.

[72] Chalk‐Wilayto J , Ossi‐Lupo K , Raguet‐Schofield M . 2016 Growing up tough: Comparing the effects of food toughness on juvenile feeding in Sapajus libidinosus and *Trachypithecus phayrei* crepusculus. J Hum Evol 98:76–89. 27544691 10.1016/j.jhevol.2016.07.004

[73] Lazzari V , Guy F . 2014 Quantitative three‐dimensional topography in taxonomy applied to the dental morphology of catarrhines. Bull Mem Soc Anthr 26:140–146.

[74] Prufrock KA , López‐Torres S , Silcox MT , Boyer DM . 2016 Surfaces and spaces: Troubleshooting the study of dietary niche space overlap between North American stem primates and rodents. Surf Topogr Metrol Prop 4:024005.

[75] Prufrock KA , Boyer DM , Silcox MT . 2016 The first major primate extinction: An evaluation of paleoecological dynamics of North American stem primates using a homology free measure of tooth shape. Am J Phys Anthropol 159:683–697. 26739378 10.1002/ajpa.22927

[76] Evans AR , Janis CM . 2014 The evolution of high dental complexity in the horse lineage. Ann Zool Fenn 51:73–79.

[77] Yamashita N et al. 2015 Mechanical food properties and dental topography differentiate three populations of *Lemur catta* in Southwest Madagascar. J Hum Evol 98:66–75. 10.1016/j.jhevol.2015.09.006. 26601707

[78] Berthaume MA . 2013 Tooth cusp radius of curvature as a dietary correlate in primates. Doctoral Dissertation, University of Massachusetts Amherst p 1–184.

[79] Maas MC , Dumont ER . 1999 Built to last: The structure, function, and evolution of primate dental enamel. Evol Anthropol 8:133–152.

[80] Constantino PJ , Lee JJW , Chai H , et al. 2010 Tooth chipping can reveal the diet and bite forces of fossil hominins. Biol Lett 6:826–829. 20519197 10.1098/rsbl.2010.0304 PMC3001363

[81] Venkataraman VV , Glowacka H , Fritz J , et al. 2014 Effects of dietary fracture toughness and dental wear on chewing efficiency in geladas (*Theropithecus gelada*). Am J Phys Anthropol 155:17–32. 25043998 10.1002/ajpa.22571

[82] Laird MF , Vogel ER , Pontzer H . 2016 Chewing efficiency and occlusal functional morphology in modern humans. J Hum Evol 93:1–11. 27086052 10.1016/j.jhevol.2015.11.005

[83] Lucas PW , Luke DA . 1983 Methods for analyzing the breakdown of food in human mastication. Arch Oral Biol 28:813–819. 6579911 10.1016/0003-9969(83)90037-7

[84] Lucas PW , Corlett RT , Luke DA . 1986 Postcanine tooth size and diet in anthropoid primates. Z Morphol Anthropol 76:253–276. 3101303

[85] Berthaume M et al. 2010;The effect of early hominin occlusal morphology on the fracturing of hard food items. Anat Rec 293:594–606. 10.1002/ar.21130 20235316

[86] Berthaume MA , Dumont ER , Godfrey LR , Grosse IR . 2013 How does tooth cusp radius of curvature affect brittle food item processing? J R Soc Interface 10:20130240. 23635495 10.1098/rsif.2013.0240 PMC3673162

[87] Evans AR , Sanson GD . 1998 The effect of tooth shape on the breakdown of insects. J Zool 246:391–400.

[88] Freeman PW , Lemen CA . 2006 Puncturing ability of idealized canine teeth: edged and non‐edged shanks. J Zool 269:51–56. 10.1111/j.1469-7998.2006.00049.x.

[89] Xie Y , Hawthorne H . 2002 Effect of contact geometry on the failure modes of thin coatings in the scratch adhesion test. Surf Coatings Technol 155:121–129.

[90] Berthaume MAMA et al. 2014 The effects of relative food item size on optimal tooth cusps sharpness during brittle food item processing. J R Soc Interface 11:20140965. 25320068 10.1098/rsif.2014.0965 PMC4223924

[91] Hlusko LJ et al. 2016 The integration of quantitative genetics, paleontology, and neontology reveals genetic underpinnings of primate dental evolution. Proc Natl Acad Sci 113:9262–9267. 27402751 10.1073/pnas.1605901113 PMC4995955

[92] Townsend G , Bockmann M , Hughes T , Brook A . 2012 Genetic, environmental and epigenetic influences on variation in human tooth number, size and shape. Odontology 100:1–9. 22139304 10.1007/s10266-011-0052-z

[93] Häkkinen TJ , Sova SS , Corfe IJ , Tjäderhane L , Hannukainen A , Jernvall J . 2019 Modeling enamel matrix secretion in mammalian teeth. PLOS Comput Biol 15:e1007058. 31141513 10.1371/journal.pcbi.1007058 PMC6541238

[94] Stojanowski CM , Paul KS , Seidel AC , Duncan WN , Guatelli‐Steinberg D . 2019 Quantitative genetic analyses of postcanine morphological crown variation. Am J Phys Anthropol 168:606–631. 30747449 10.1002/ajpa.23778

[95] Skinner MM , Evans A , Smith T , et al. 2010 Brief communication: Contributions of enamel‐dentine junction shape and enamel deposition to primate molar crown complexity. Am J Phys Anthropol 142:157–163. 20091839 10.1002/ajpa.21248

[96] Selig KR et al. 2018 First 3D dental topographic analysis of the enamel‐dentine junction in non‐primate Euarchontans: Contribution of the enamel‐dentine junction to molar morphology J Mamm Evol. 26:587–598.

[97] Godfrey LR , Winchester JM , King SJ , Boyer DM , Jernvall J . 2012 Dental topography indicates ecological contraction of lemur communities. Am J Phys Anthropol 148:215–227. 22610897 10.1002/ajpa.21615

[98] Henry AG et al. 2012 The diet of *Australopithecus sediba* . Nature 487:90–93. 22763449 10.1038/nature11185

[99] Marshall AJ , Wrangham RW . 2007 Evolutionary consequences of fallback foods. Int J Primatol 28:1219–1235.

[100] Dominy NJ , Vogel ER , Yeakel JD , Constantino P , Lucas PW . 2008 Mechanical properties of plant underground storage organs and implications for dietary models of early Hominins. Evol Biol 35:159–175.

[101] Yamagiwa J , Basabose AK . 2009 Fallback foods and dietary partitioning among Pan and Gorilla. Am J Phys Anthropol 140:739–750. 19890854 10.1002/ajpa.21102

[102] Kuroda S , Nishihara T , Suzuki S , Oko RA . 1996 Sympatric chimpanzees and gorillas in the Ndoki Forest, Congo In: Marchant LF, Nishida T, editors. Great Apes Societies, Cambridge: Cambridge University Press p 71–81.

[103] Boyer DM , Costeur L , Lipman Y . 2012 Earliest record of Platychoerops (primates, Plesiadapidae), a new species from Mouras quarry, Mont de Berru, France. Am J Phys Anthropol 149:329–346. 22926965 10.1002/ajpa.22119

[104] Pampush JD , Spradley JP , Morse PE , et al. 2018 Adaptive wear‐based changes in dental topography associated with atelid (Mammalia: Primates) diets. Biol J Linn Soc 124:584–606.

[105] Swindler DR . 2002 Primate dentition: an introduction to the teeth of non‐human primates, Cambridge: Cambridge University Press.

[106] Kamilar J , Pokempner A . 2008 Does body mass dimorphism increase male–female dietary niche separation? A comparative study of primates. Behaviour 125:1211–1234.

[107] Cerling TE , Mbua E , Kirera FM , et al. 2011 Diet of Paranthropus boisei in the early Pleistocene of East Africa. Proc Natl Acad Sci U S A 108:9337–9341. 21536914 10.1073/pnas.1104627108 PMC3111323

[108] Ungar PS , Grine FE , Teaford MF . 2008 Dental microwear and diet of the Plio‐Pleistocene hominin Paranthropus boisei. PLoS One 3:e2044. 18446200 10.1371/journal.pone.0002044 PMC2315797

[109] Swindler DR . 2002 Fourth molars in anthropoidea. Dent Anthropol 16:26–28.

[110] Schroer K , Wood B . 2015 The role of character displacement in the molarization of hominin mandibular premolars. Evolution 69:1630–1642. 25913032 10.1111/evo.12672

[111] Lazzari V , Charles C , Tafforeau P , Vianey‐Liaud M , Aguilar JP , Jaeger JJ , Michaux J , Viriot L 2008 Mosaic convergence of rodent dentitions. Stajich JE, editor. PLoS One 3:e3607. 18974837 10.1371/journal.pone.0003607 PMC2572836

[112] Klukkert ZS et al. 2012 Dental topographic analysis of the molar teeth of primates In: Bell LS, editor. Forensic Microsc Skelet Tissues, NJ: Humana Press p 145–152. 10.1007/978-1-61779-977-8_9 22907407

